# Multi-Compartmentalisation in the MAPK Signalling Pathway Contributes to the Emergence of Oscillatory Behaviour and to Ultrasensitivity

**DOI:** 10.1371/journal.pone.0156139

**Published:** 2016-05-31

**Authors:** Aban Shuaib, Adam Hartwell, Endre Kiss-Toth, Mike Holcombe

**Affiliations:** 1 Department of Infection, Immunity and Cardiovascular Disease, University of Sheffield, Sheffield, United Kingdom; 2 Department of Computer Science, University of Sheffield, Sheffield, United Kingdom; 3 INSIGNEO Institute for in silico Medicine, University of Sheffield, Sheffield, United Kingdom; 4 Department of Automatic Control and Systems Engineering, University of Sheffield, Sheffield, United Kingdom; 5 Advanced Computing Research Centre, The Sheffield Bioincubator, University of Sheffield, Sheffield, United Kingdom; Florida State University, UNITED STATES

## Abstract

Signal transduction through the Mitogen Activated Protein Kinase (MAPK) pathways is evolutionarily highly conserved. Many cells use these pathways to interpret changes to their environment and respond accordingly. The pathways are central to triggering diverse cellular responses such as survival, apoptosis, differentiation and proliferation. Though the interactions between the different MAPK pathways are complex, nevertheless, they maintain a high level of fidelity and specificity to the original signal. There are numerous theories explaining how fidelity and specificity arise within this complex context; spatio-temporal regulation of the pathways and feedback loops are thought to be very important. This paper presents an agent based computational model addressing multi-compartmentalisation and how this influences the dynamics of MAPK cascade activation. The model suggests that multi-compartmentalisation coupled with periodic MAPK kinase (MAPKK) activation may be critical factors for the emergence of oscillation and ultrasensitivity in the system. Finally, the model also establishes a link between the spatial arrangements of the cascade components and temporal activation mechanisms, and how both contribute to fidelity and specificity of MAPK mediated signalling.

## Introduction

Cells constantly receive external signals reflecting changes in their environment, which they should respond to accordingly. An array of signal transduction pathways and signalling mechanisms have evolved that translate these external cues into specific cellular responses. One of these central intracellular signalling pathways is known as the mitogen activated protein kinase (MAPK) pathway [1].

**Fig 1:**
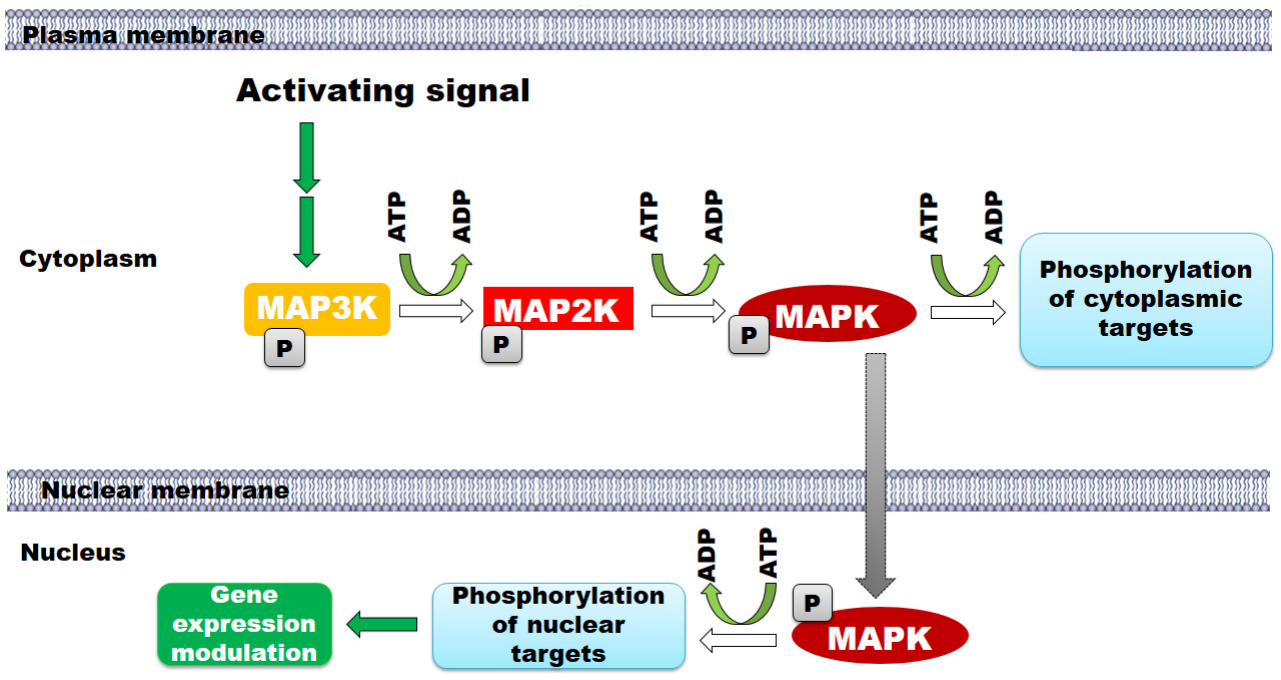
A schematic representation of the MAPK cascade and its activation mechanisms. The MAPK pathway is composed of three levels. The signal is transduced through phosphorylation events where mitogen activated protein kinase kinase kinase (MAP3K, also known as MAPKKK) phosphorylates mitogen activated protein kinase kinase (MAP2K, also known as MAPKK) leading to its activation and thus the phosphorylation and activation of the mitogen activated protein kinase (MAPK). Active MAPK phosphorylates protein targets in the cytoplasm and the nucleus. For mediating nuclear events MAPK translocates to the nucleus where it phosphorylates many proteins, which control gene expression.

The pathway is a three-tiered cascade involving three enzymes, the MAPK kinase kinase (MAPKKK), the MAPK kinase (MAPKK) and the MAPK. Mechanistically, pathway activation relies on the propagation of phosphorylation events downstream of the cascade [2, 3] as shown in Fig 1. The MAPK pathway plays a critical role in cells as it regulates numerous and diverse cellular responses [4–6], including regulation of the cell cycle, influencing differentiation, survival and apoptosis. Historically, these responses were attributed to distinct MAPK pathways, mediating a specific response [7–10]. Three groups of MAPKs have been characterised and were initially thought to respond to distinct signals. These include the ERK, JNK and p38 kinases; each of these is a “common name” for groups of highly similar proteins, encoded by small gene families. However, as the interest and knowledge in the molecular mechanisms that control these pathways grew, two issues have emerged: (i) a single pathway is capable of mediating opposing effects as seen with extracellular signal-regulated kinase (ERK) mediating either the differentiation or the division of PC12 cells [11, 12] (ii) Some of the responses the pathways triggered can overlap, with different MAPKs converging to mediate the same cellular responses in the same cell [13–15]. Furthermore, accumulating evidence showed that the MAPK pathways function as a network connected at different levels of the kinase cascade. Nonetheless, given this complexity, cells maintain high fidelity to the initial signal and respond efficiently. It is believed that properties arise from the activation behaviour of the pathway such as the signal magnitude, ultrasensitivity and oscillation. These thought to be influenced by the spatial and temporal aspects of MAPK pathway activation.

Temporal regulation of the MAPK pathway affects the cascade’s dynamics. It is also thought that the signal dynamics such as the magnitude of the response, duration and oscillation play a role in specifying the cellular outcome. For instance, it was long reported that sustained and transient activation of ERK caused quiescence and proliferation, respectively in Swiss 3T3 cells [16], PC12 and yeast cells [17]. In addition, high response magnitude enabled cell arrest while moderate magnitude had facilitated proliferation as seen in mouse embryonic fibroblasts (MEFs) [18, 19]. Oscillation in particular is thought to play a significant role in facilitating the specificity of the signal as the frequency and amplitude of the waves could encode for specific aspects of both gene transcription and translational changes. Oscillation is thought to emerge from regulatory mechanisms, which modulate the cascade input and output. Oscillations were observed previously in calcium signalling and in the NF-κB pathway; however, this was only recently reported in the MAPK pathway [20, 21]. Nevertheless, oscillation in the MAPK pathway was predicted and demonstrated before using *in silico* models [22]. These models had proposed that regulatory machineries may involve feedback loops. The majority of the models had shown that negative feedback loops are chiefly responsible for the emergence of the oscillatory behaviour. Some models also propose that the interplay between positive and negative feedback is fundamental to generate signals that code for specific responses [23–26]. These oscillatory behaviours are suggested to be responsible for allowing the cell to choose to proliferate, go into senescence or differentiate. Some suggest that they may play a role in synchronising the responses of multiple cells to a signal mirroring the circadian rhythm [27].

The spatial distribution of the MAPK pathway is critical to generating specific responses. The first indications for this were coming from contrasting responses observed between nuclear and cytoplasmic ERKs triggered by the same stimulus. In fibroblasts and embryonic carcinoma cells, ERK activation and nuclear translocation caused proliferation. However, by preventing ERK translocation these cells became senescent and differentiated, respectively [28, 29]. An impact of spatial distribution was also seen during the activation of the beta-adrenergic receptors, which transiently activated ERK upon stimulation, which then translocated to the nucleus to regulate gene-expression. However, with the internalisation of receptors to the endosomal compartment, ERK activation becomes sustained and its action is confined to the cytosol. Also, Teis *et al*. have shown that there are separate pools of ERK in the plasma membrane and the endoplasmic reticulum and both of them mediate distinct actions. Depleting the endoplasmic reticulum (ER)-ERK pool led to an altered activation/inactivation dynamics of the pathway. Once the endoplasmic reticulum (ER)-ERK pool was demolished/decreased the effect disappeared and only returned with the re-introduction of the ER-ERK pool [30, 31]. Furthermore, in neuronal cells, the discrimination between the epidermal growth factor (EGF) and nerve growth factor (NGF) signalling is also thought to be due to the different compartments ERK resides in. Distinctive cellular responses were also observed when MAPKKs were localised in different cellular compartments [32]. All of the above examples point to the critical role of compartments and spatial separation in mediating specific responses of the MAPK pathway.

In the work reported here, we were interested in characterising the interaction between spatial and temporal parameters in the MAPK cascade and how these influence pathway activity. We approached this by using an agent-based computer modelling approach, whereby every key molecule and compartment were explicitly modelled. This high level of detailed modelling provided an innovative basis for examining the role that compartmentalisation plays in MAPK activation. The main purpose of the model was to explain why compartmentalisation is necessary in order to achieve the various behaviours seen in biology. Less detailed modelling approaches are unlikely to be as informative.

We characterised the effect of compartmentalisation on MAPK activation and how it influences the formation of phosphorylated MAPK, thereby providing a novel insight as the issue of multi-compartmentalisation has not previously been highly addressed by *in silico* models of the cascade. We compared two types of models; a two-compartment model (which commonly used to study the cascade) and a novel, multi-compartment model. Our model shows that multi-compartments play an important role in the emergence of oscillatory behaviour in the MAPK cascade. In addition, we infer from the data that the balance between inhibitory and activating inputs at the level of the MAPKK is critical for the appearance of oscillation in the system. Our ABM model suggests a fruitful strategy of integrating spatial and temporal regulation of the MAPK pathway and their influence on oscillation, and thus on signal specificity and efficiency.

## Results

### Agent Based Models of MAPK Activation

**Fig 2:**
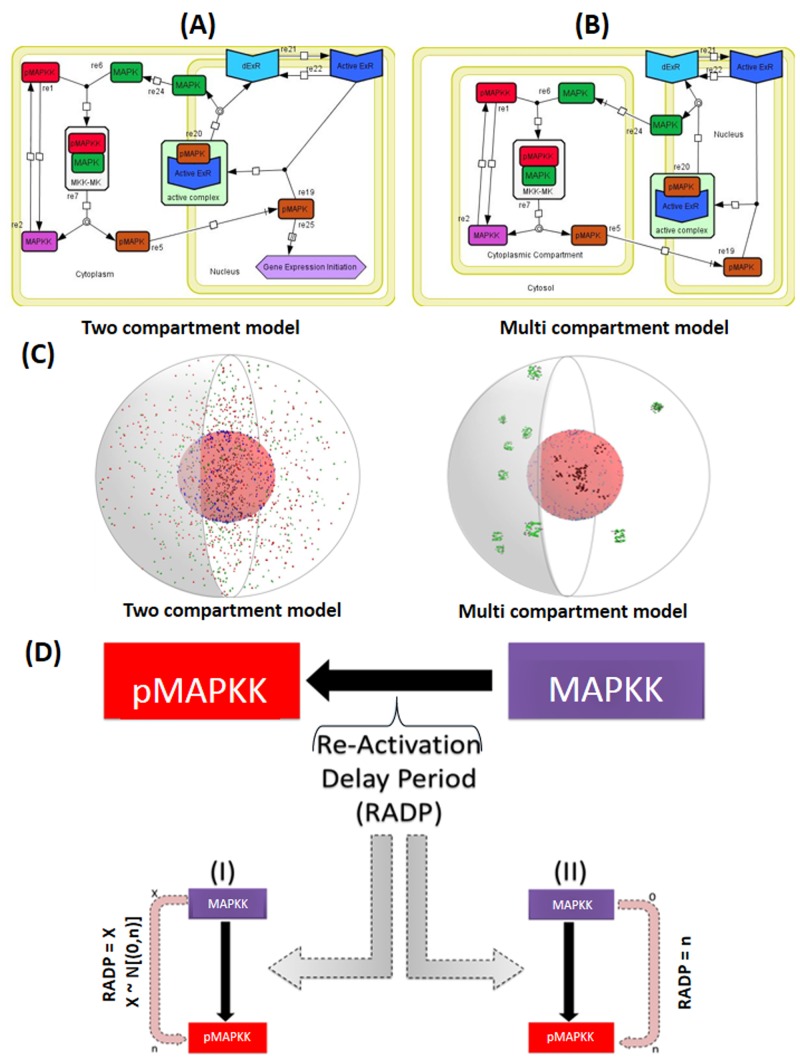
Graphical representation of cytoplasmic and nuclear events in the two-compartment and multi-compartment agent-based models (ABMs). The basic two dimensional design of the two and multi-compartment models of the two tier MAPK pathway represented using Systems Biology Graphical Notation (SBGN) standard annotations. (A) Illustrates the design of the two-compartment ABM whilst (B) describes the design of the multi-compartment ABM. Details of the two model design, structure and functionality are provided in the Materials and Methods section. (C) A three-dimensional (3D) visualisation of both the two-compartment vs. the multi-compartment model. The right hand side of both 3D representations is a 3D cross section of the “cell”. The cytoplasm is represented by the grey space around the nucleus. Inside the cytoplasm green spheres are MAPK, red spheres are pMAPKK, violet spheres are MAPKK, within the nuclear space, black spheres are pMAPK agents, dark blue are ExRs and light blue are dExRs. (D) Modelling the Re-Activation Delay Period (RADP) in the ABM: once pMAPKK agents change state into MAPKKs, they become dormant for period of time, and once this dormancy period is passed MAPKKs are re-activated. RADP was modelled either stochastically (I) or deterministically/periodically (II). In the stochastic model (I), RADP (X) was generated randomly for every individual pMAPKK agent, where X was a value between 0 and the chosen maximum value n (X ~ N ([0, n])). Periodic RADP was always identical for every MAPKK formed (RADP = n).

We have constructed two models of the MAPK pathway in order to address the effect of compartmentalisation of the MAPK components on pathway activation (Fig 1). The first model mimicked a two-compartment system, including the cytoplasm and the nucleus. The second model incorporated a multi-compartment system including the nucleus (with identical properties as compared to the two compartment model), cytoplasm, and ten randomly located cytoplasmic compartments. The two models share a number of common features. They both rely on binding events as the key factor to drive them. Agents move spontaneously and follow Brownian motions with few restrictions (read the agents descriptions in Methods). Both models are set and constructed in a three dimensional spherical cell as shown in Fig 2C. All agents cycle between activated and deactivated states, all the MAPKK are subjected to deactivating inputs (mainly RADP) and there is no loss of agents or re-creation of agents in the system. The working mechanisms of both models are equivalent. Briefly, pMAPKK activates MAPK leading to the formation of pMAPK, which translocates to the nucleus. Once translocated to the nucleus, MAPK could interact with active exporting receptors (ExR) and removed from the nucleus (Fig 2A and 2B). Alternatively, pMAPK can interact with an active transcription factor, which triggers MAPK-dependent gene expression.

Simple rules were assigned to the agents in both models (S1 File). These rules specified the agents’ movement and the manner in which they interacted amongst themselves and with their environment. The execution of the rules depends on the functions assigned to the agents and the agents’ memory. Agents’ memories are stored and regularly updated with every state transition of the agents and with every model iteration. A list of the memory components, messages and functions of each agent are listed in S1 File.

Communication between the agents was achieved by the use of messages. The messages were inputted and outputted using the agents’ functions. The messages were stored in the message board (Libmboard) and each agent accessed and read messages needed for the interaction with its interacting partner. Agents went through state transitions and the memory parameters were updated once the messages were read and the functions were performed. The physical interaction between the agents and the different agent states (DAS) were determined by assigning an interaction value. Once the interacting agents and the DAS were within the specified proximity, interaction between the agents and/or DAS occurred.

We also examined the effect of pMAPKK availability for the interaction with MAPK and how these also influence the dynamics of pathway activation. Two scenarios were modelled by introducing the parameter re-activation delay period (RADP, Fig 2D): an activation by strong stimulus *vs* weak inhibition of the signal at the level of MAPKK (when RADP < 15 min) and activation by a weak stimulus *vs* a strong signal inhibition at the level of MAPKK (when RADP > 15 min). Further details on RADP will be discussed below.

### Calibration of the ABM

**Fig 3:**
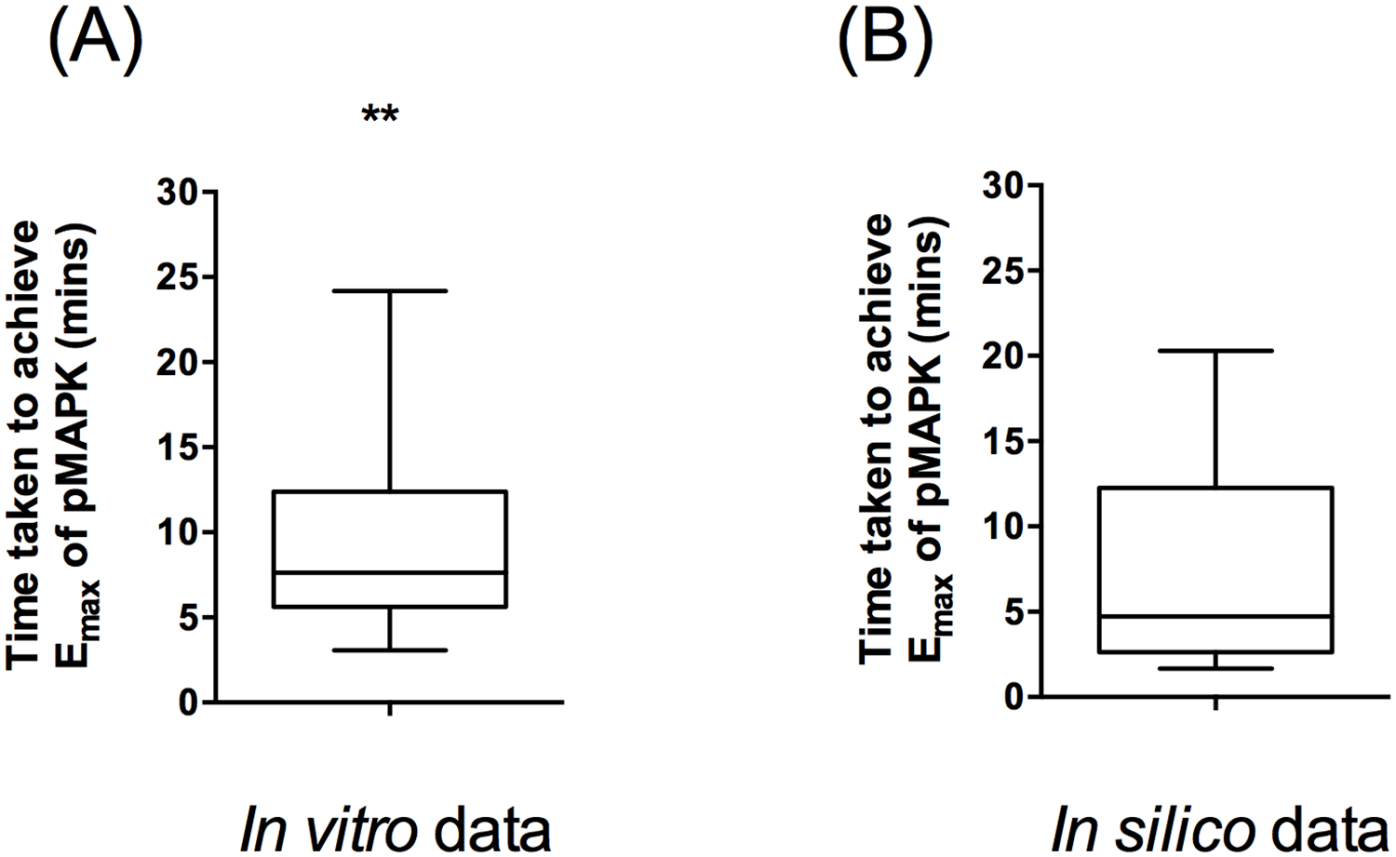
Analysis of MAPK activation dynamics observed *in vitro* in the published literature. 84 MAPK activation dynamics values were collected from published literature and the time to achieve E_max_ were plotted. A whisker plot with the median values are presented. (A) 63 *in vitro* data points from the analysed data were selected, plotted and analysis for normality was conducted. D'Agostino & Pearson omnibus normality test showed that the *in vitro* data for MAPK activation dynamics were not normally distributed (**: p<0.01). (B) 21 *In silico* data-points were extracted from the above literature and normality analysis was performed as for (A), demonstrating that the *in silico* data was normally distributed.

A critical parameter of pathway activation dynamics is the time to elicit E_max_ of MAPK activation. In order to calibrate our ABM model, 63 experimental data points from 34 publications reported on MAPK activation time (E_max_) were extracted from the published literature (S1 File) and analysed as shown in Fig 3A. The statistical analysis of this data revealed that the values were not normally distributed Fig 3A. In contrast, 21 E_max_ values from *in silico* models within these studies were normally distributed (Fig 3B). Therefore, the median time for maximal activation (7.63 min) was calculated from the experimental dataset and used to calibrate our model and to convert the time-step in the ABM into time values.

### Sensitivity Analysis of the ABM

**Fig 4:**
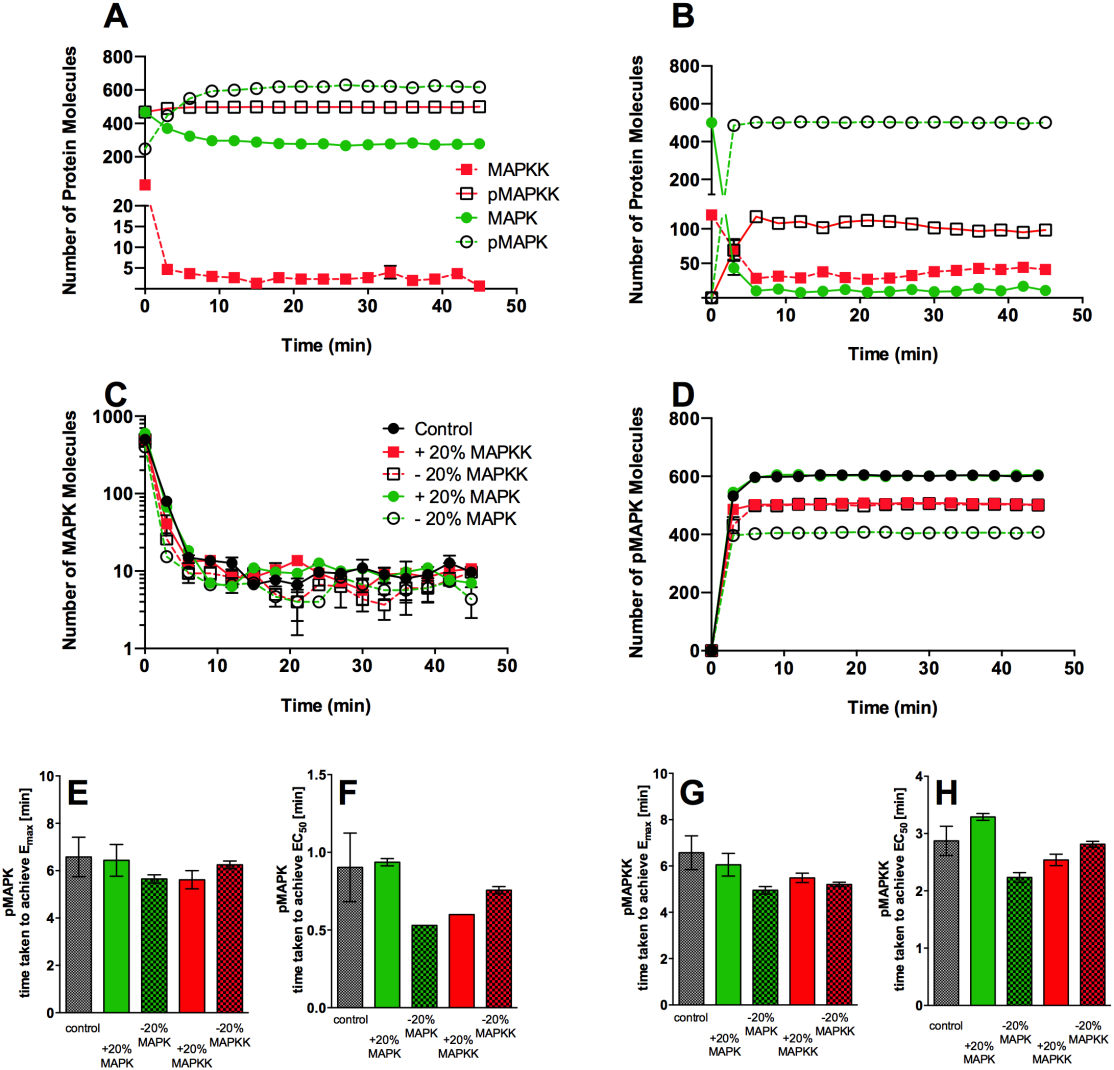
Robustness and sensitivity analysis of the ABM models. The basic two compartment (A) and multi-compartment models (B) were run multiple times (n = 3). (A) The graph shows a run of the complete two compartment model in the presence of constitutively active MAPKK agents and the emergent kinetic behaviour of pMAPK and MAPK agents. The graph shows the interaction between pMAPKKs and MAPKs until the level of pMAPKs and MAPKs plateau as the interaction reaches equilibrium. The pattern emerging is a graded ultrasensitive response (whereby E_max_ ≥ 10 min). The model shows rapid activation of MAPK and formation of pMAPK but does not show ultrasensitive behaviour. (B) A graph generated from the multi-compartment model without a constitutively active MAPKK, this model shows that the multi-compartment system is capable of generating high level of pMAPK within a short period of time and with a gradual activation of MAPKK agents in addition to demonstrating an ultrasensitive behaviour. Individual data points for each run and the mean of the values are plotted. (C-H) Sensitivity analysis of the multi-compartment ABM to examine model sensitivity to manipulation of initial agent numbers. The number of each agent was altered by ±20%, compared to the control model. The number of pMAPK (E, F) and pMAPKK (G, H) agents were plotted. Time to achieve both E_max_ and EC_50_ were determined under each condition and the analysis of variance (ANOVA) was used to test for statistically significant changes. The analyses showed no significant difference between the different ABM conditions.

We tested the robustness of our models similar to approaches reported in previous studies of modelling MAPK signalling [33]. First, the two-compartment and multi-compartment models were run multiple times (n = 3) and the number of each species of agents were plotted at set time points for the individual runs (Fig 4A and 4B). Analysis of the standard deviation of the active MAPK species (pMAPK and pMAPKK) demonstrated that the models were robust. Whilst SD in the two-compartment model was low for both pMAPKK and pMAPK (SD pMAPK <3.3%, SD MAPKK <2%), SD for pMAPKK in the multi-compartment model were greater (1.5–37%). However, SD for pMAPK was <2.5% at every time point, suggesting that such variation in pMAPKK levels is “tolerated” by the system, leading to a highly robust pathway activation. Next, the number of initial MAPKK and MAPK agents have been altered by 20% in the multi-compartment model, and MAPK and pMAPK agent numbers were plotted at set time points in three consecutive runs (Fig 4C and 4D). Variation between runs at each time point was < 5%, further suggesting that our models were robust. Finally, the impact of altered MAPK or MAPKK levels on the dynamics of MAPK activation was analysed. Time to achieve pMAPK E_max_ and EC_50_ were determined in each of the models and conditions in Fig 4A–4D and one-way ANOVA was used to establish the impact of altered initial MAPK and MAPKK levels on the generation of pMAPK and pMAPKK (Fig 4E–4H). In short, alteration of MAPKK and MAPK levels did not affect MAPK and MAPKK activation dynamics, further supporting that our model was robust and insensitive to up-to 40% in initial agent numbers.

### Compartmentalisation Is Responsible for the Rapid Responsiveness of the MAPK System

We implemented two models to investigate the effect of compartmentalisation on MAPK pathway activation. In the initial, two-compartment model (as described in the Methods), the MAPK and MAPKK agents were moving freely in the cytoplasm. We investigated the dynamics of the formation of the pMAPK in this model with the ratio between MAPKK and MAPK set at 1:1 and MAPKK being in a constitutively active state; this was to reflect a strong and sustained activation signal, similar to the oocyte system Ferrel investigated as a model of irreversible pathway activation [34]. As shown in Fig 4A, the levels of activated MAPKK in the system hardly changed over time in this configuration, resulting in a sharp formation of pMAPK and a rapid achievement of equilibrium. However, an initial lag-period of pMAPK accumulation (≈94 seconds (s)) was observed. Interestingly, an equilibrium of 1:2 MAPK to pMAPK ratio was established in the two-compartment model, different from ordinary differential equation (ODE) models that are based on Ferrel’s original MAPK pathway model.

Next, a multi-compartment model was constructed to elucidate the impact of spatially restricted MAPKK/MAPK complexes on the dynamics of pMAPK formation. To simulate physiological conditions of resting cells in this model, the majority (95%) of the MAPKK agents were not active and the majority of the MAPK agents were not phosphorylated/activated initially. A model included an activation signal at 0 time point with MAPKK remaining active; this resulted in a system which was highly sensitive to activation with a rapid rate of pMAPK formation (≈ 11.5 ± 0.4% of MAPK was converted to pMAPK per min), and thus a rapidly reaching equilibrium. In addition, in the multi-compartment model 98 ± 0.2% of MAPK was converted into pMAPK and translocated to the nucleus (Fig 4B). In contrast, the two-compartment model had generated a less sensitive system, where only ≈ 82.4% ± 0.2% of MAPK molecules were converted to pMAPK per min. Furthermore, levels of pMAPK generated were lower in the two compartment model and only a 70.3 ± 2.2% reduction in cytoplasmic MAPK levels once the system had fully triggered (Fig 4A).

However, due to the constitutive activity of MAPKK, particularly in the multi-compartment model, the levels of MAPK did not return to the initial values. Thus we modified our model to address this and describe its results below.

### MAPKK Re-Activation Delay Influence Dynamics of pMAPK Formation in a Multi-Compartment Model

In cells, activated MAPKK is deactivated by phosphatases [35, 36]. Thus the balance between activation and inactivation relies on the number of active pMAPKK molecules *versus* inactive MAPKKs, which is influenced by the rate of phosphatase activity. To address this issue in the ABM model, a re-activation delay for MAPKK was introduced. Once pMAPKK interacts with MAPK it enters a dormant state where it is not capable of activating MAPK and this period of inactivity is defined as the re-activation delay period (RADP). The effect of re-activation delay was modelled deterministically and stochastically. Initially, different RADPs were investigated and systems behaviour in stochastic vs. deterministic models were compared. Stochasticity of RADP values were analysed, employing one-sample runs test. First, RADP values were collected for five independent MAPKK agents during a model run. Secondly, RADP values were collected for the same MAPKK agent during four independent runs of the model (individual RADP values are presented in S3 File). In both scenarios, the one-sample runs test yielded p>0.05 for every agent/run, demonstrating that RADP values were stochastic.

**Fig 5:**
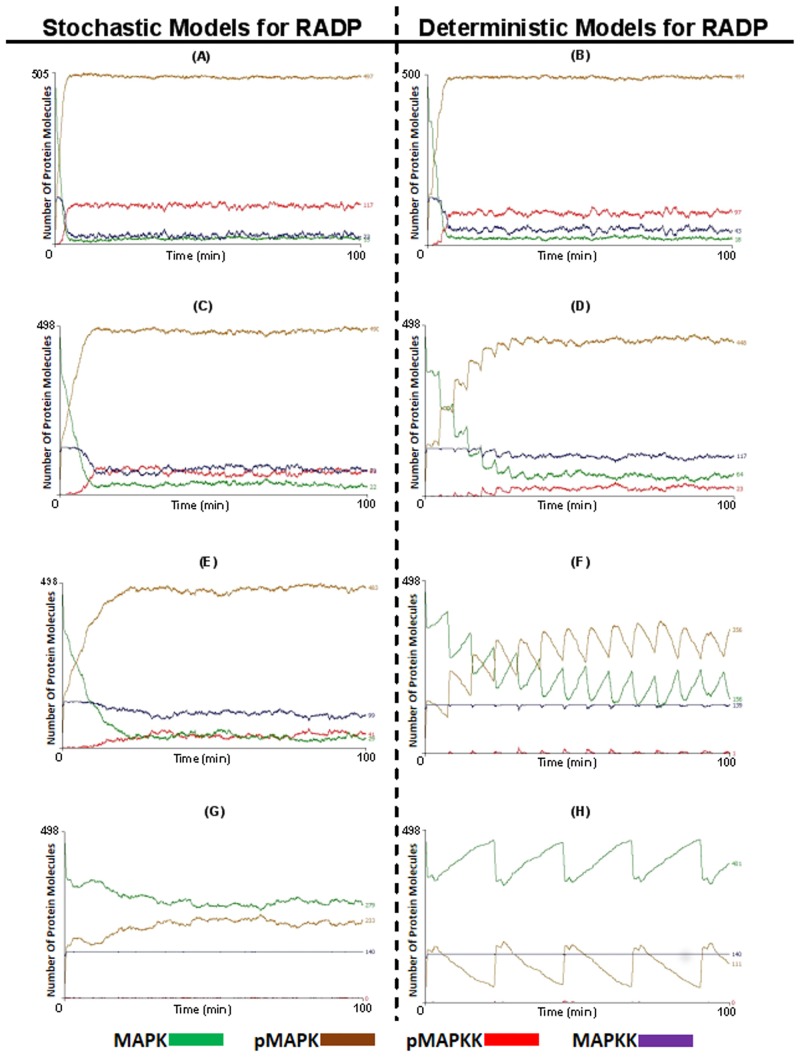
The effect of delaying MAPKK re-activation on the dynamics of MAPK activation and MAPKK levels. Once pMAPKK agents bind and activate MAPKs to pMAPKs, pMAPKKs convert to a dormant state (MAPKK). The length of this dormancy period was set and its effects on the levels of pMAPK, MAPK, pMAPKK and MAPKK were monitored. In (A) and (B) the re-activation delay period (RADP) was set at a short period (0 ≤ RADP ≤ 90 s), while in (C) and (D) RADP was set to an intermediate period (0 ≤ RADP ≤ 4.53 min); in (E) and (F) RADP was set to a the highest range of the intermediate period (0 ≤ RADP ≤ 7.55 min); while in (G) and (H) RADP was set to long periods (0 ≤ RADP ≤ 22.6 min). The figures on the left hand side were stochastic (where the RADP was set stochastically within the specified delay period every time pMAPKK switched state to MAPKK); while models on the right hand side were deterministic (where MAPKK returns to the active pMAPKK state after a fixed period.

At lower RADPs (0 ≤ RADP < 90 (s)) the MAPK system retained its rapid activation rate and high level of pMAPK formed in both deterministic and stochastic models (Fig 5A *vs*. 5B). In contrast, at slightly longer RADPs, the deterministic model showed graded responses during the initial activation phase (Fig 5B *vs*. 5D). These graded responses were also observed in the stochastic models with minimum stochasticity (for instance, 4.38 ≤ RADP < 4.53 min, S1 Fig); hence the models closely resembled the deterministic models.

Models with longer RADPs and at maximum stochasticity (0 ≤ RADP < 7.55 min) generally retained their ability to generate high levels of pMAPK (93.9 ± 1.7% reduction of MAPK levels at E_max_ compared to t_0_, S2A Fig), though the rate of activation decreased and the time to achieve E_max_ increased from 6.24 ± 1.3 min to 26.7 ± 6.9 min (Fig 5E and S2B Fig). However, if the RADP was fixed to create a deterministic model (RADP = 7.55 min) or one with minimal stochasticity (7.53 ≤ RADP < 7.55 min), the graded responses observed earlier evolved into an oscillatory behaviour (Fig 5F).

In a stochastic model of RADP, when the RADP was >15 min and when E_max_ was reached, the levels of inactivated MAPK had fallen by 47.4 ± 3.9% (from 100% at t_0_ and compared to ~95% reduction in MAPK levels observed in the other models we presented here, S2A Fig). Although this is a significant reduction, the levels of MAPK were still higher than the EC_50_, and did not reach 5% of MAPK levels at t_0_ (Fig 5G). Nonetheless, this model still demonstrated a level of responsiveness, which had arisen from the ability of extremely low levels of MAPKK agents to maintain a high level of pMAPK in the model. In contrast, the deterministic models with RADPs higher than 15 min, the graded dynamics of pMAPK formation evolve into sustained oscillatory behaviour (Fig 5H).

In these multi-compartment models of re-activation delay, although increasing the RADP led to lower steady state pMAPK levels (Fig 5F–5H) and reduced MAPK: pMAPK ratio, neither of them were capable of re-establishing the levels of MAPK and pMAPK at t_0_. Nonetheless, in deterministic models, t_0_ MAPKK levels were re-established once RADP was set at > 7.55 min. On the other hand, this behaviour was only seen at long RADP in the highly stochastic models (data not shown).

### Alterations in RADP Fail to Display an Oscillatory Behaviour and to Regulate pMAPK Formation Dynamics in a Two-Compartment Model of the MAPK Cascade

**Fig 6:**
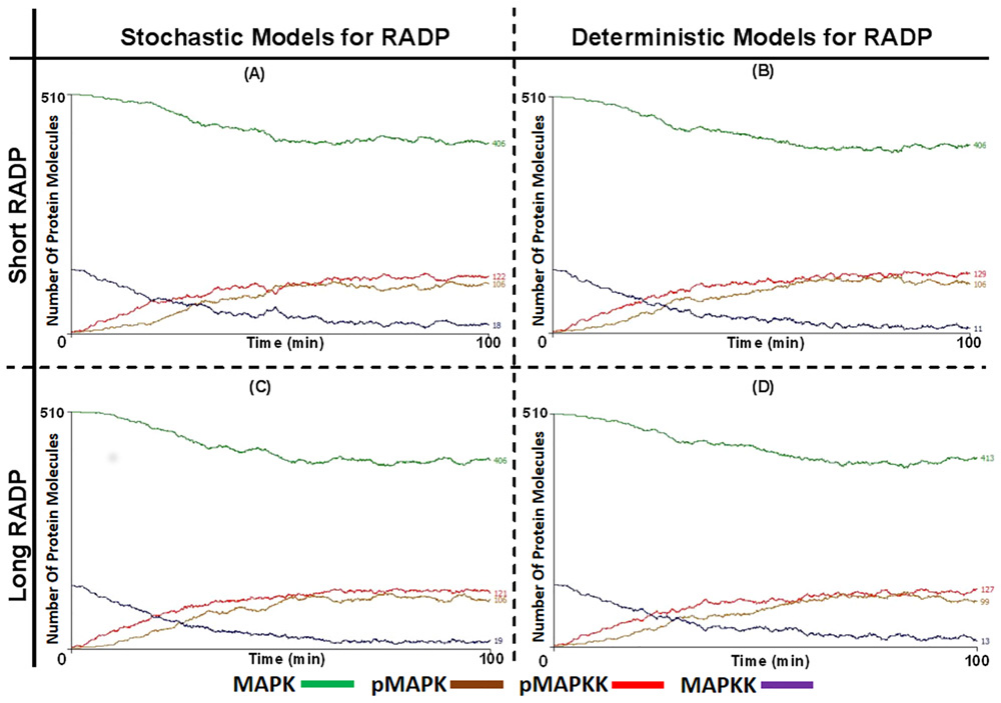
The effect of MAPKK re-activation delays on the dynamics of pMAPK formation and pMAPKK levels in two-compartment system. The re-activation delay characteristics of pMAPKK (red) were applied to the two-compartment ABM and the effects were monitored. Initially the effect of a deterministic versus a stochastic model were looked at. In (A) and (B) short RADPs (0 ≤ RADP ≤ 90 s) were tested, (A) was the model with stochastic RADP while (B) was the model with deterministic/periodic RADP. There was no significant difference between the graphs generated by either ABMs when the analysis of variance (ANOVA) was used. However, both of the models had generated lower activation rate and formation of pMAPK (brown) and pMAPKK (violet) in comparison to the multi-compartment system. The graphs in (C) and (D) were generated with long RADPs (0 ≤ RADP ≤ 22.6 min), pMAPK formation, pMAPKK and MAPK (green) activations patterns were similar to those with short RADP seen in (A) and (B). Unlike multi-compartment models, deterministic models with intermediate or long RADPs did not generate any oscillatory pattern.

Next, the re-activation delay characteristics of MAPKK were tested in the two-compartmental model. In these, neither stochastic nor deterministic models of MAPKK RADP (Fig 6A, 6C and 6B, 6D respectively) produced an oscillatory behaviour for pMAPK formation dynamics and there was no significant difference between the two models with regards to pMAPK formation, MAPKK activation and re-established MAPK levels. This was seen both at RADPs ≤ 5 min (Fig 6A and 6B) and RADPs ≥ 15 min (Fig 6C and 6D). Furthermore, whilst introducing the RADP into the two compartment model did not induce oscillation, the model still maintained the characteristic graded MAPK activation dynamics for both MAPK and pMAPKK (≈60 min to reach E_max_).

Signalosome clusters have been reported previously, including lipid rafts and Ras nanoclusters [37]. In these signalling apparatus at the plasma membrane (such as rapidly accelerated fibrosarcoma1 [RAF1] and rat sarcoma [Ras]) are brought together into a very close proximity and randomly assemble and disassemble [38, 39]. This concept was applied by changing the multi-compartment model to a model with assembled signalosome clusters at the arrival of the activating signal; these clusters then disassembled and the signalosome components diffused into the cytoplasm by Brownian motion. The impact of signalosome cluster model was tested with both the deterministic and stochastic models and with long and short RADPs. This led to a two-phase response, an activation “turn on” phase and a tailing-off “shut-down” phase.

**Fig 7:**
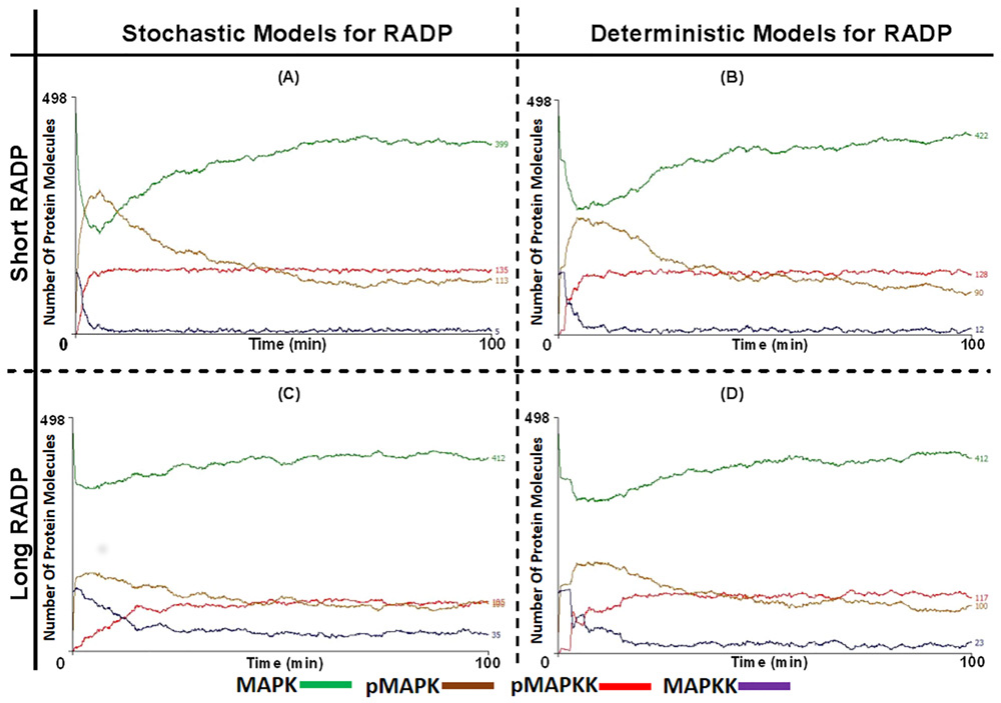
pMAPKK and pMAPK levels and rate of activation are significantly enhanced in the two compartmental model in the presence of signalosome clusters though with no significant difference between deterministic and stochastic re-activation delay (RADP) models. Deterministic and stochastic models of MAPKK RADPs were tested again in the two-compartment ABM, in the context of assembly and disassembly of pMAPKK-MAPK signalsome clusters. In both models the presence of the clusters caused a rapid rate for pMAPKK (red) activation and pMAPK formation (green). This observation shares similarity with the multi-compartment system; however, only at the initial MAPK activation stage. Yet, these cluster models differ with the multi-compartment model in three aspects; (1) the cluster model exhibits a two phase response (activation [turn on] and deactivation [turn off/recovery] phases); (2) the recovery of MAPK (seen in the post-activation phase of the signalosome cluster model) and (3) that high levels of active pMAPKK are incapable of re-establishing high levels of pMAPK. In (A) and (B) short RADPs (0 ≤ RADP ≤ 90 s) were tested, (A) was the model with stochastic RADP while (B) was the model with deterministic RADP (RADP = 90 s). The graphs in (C) and (D) were generated with long RADPs (0 ≤ RADP ≤ 22.6 min), where (C) stochastic RADP was employed while (D) deterministic RADP was utilised (RADP = 22.6 min). The dynamics of pMAPK formation, MAPKK and MAPK activations in the long RADP models were similar to those noted in the short RADP models. Student t-test no significant difference in the responses generated by stochastic and deterministic models of RADPs at long periods, except for the slightly higher pMAPKK levels in the deterministic model once the steady state was reached. This also applies to the models with short RADPs, though the stochastic models generate higher levels of pMAPK in the initial phase.

Looking at pMAPK formation dynamics as a surrogate of pathway activation, there was little difference between the stochastic (Fig 7A and 7C) and deterministic models (Fig 7B and 7D) as well as between the models using short or long RADPs (Fig 7A and 7B *vs*. Fig 7C and 7D, respectively). MAPKK-MAPK cluster formation led to a rapid accumulation of pMAPK, however, this was short lasting as MAPK levels were gradually reduced with cluster disassembly. The four models demonstrated a strong ultrasensitive response in the initial phase of activation of MAPK. However, ultrasensitive response for MAPKK was only seen in the short RADP models, while appearing to be graded in the long RADP models.

The primary differences observed between the different MAPKK-MAPK cluster models included the magnitude of pMAPK generated within the initial phase of MAPK activation. At E_max_ of the stochastic RADP model (RADP < 90 s) 60% of MAPK were activated in the initial phase. Long RADPs did not show high responsiveness and thus resulted into lower pMAPK levels and low rate for MAPK activation/pMAPK formation (33% reduction for the stochastic model and 40% for the deterministic model), though the stochastic model had shown a faster rate of pMAPK accumulation.

When assessing MAPKK activation, all models applied to this compartmental setup established maximum or near maximum pMAPKK levels at steady state, with short RADP models generating slightly higher MAPKK levels. Deterministic models of RADP, however, showed some graded responses in the initial phase of MAPKK activation (Fig 7(B) and 7(D)). Nonetheless, unlike the multi-compartment model, high levels of active MAPKK were not able to sustain high levels of pMAPK.

### The pMAPK Dynamics Obtained from the ABM Are Comparable to MAPK Dynamics Observed *In Vitro*

We looked at formation of pMAPK in the ABM model and compared it to recently published results by Shankaran *et al*. where they demonstrated the oscillation of pMAPK levels experimentally [21]. Our ABM models show a good level of correlation with their *in vitro* data, as demonstrated by statistical analysis of the dynamics of MAPK activation in the experimental vs. ABM data. Their stimulation of cells with EGF showed a temporal dynamics of pMAPK formation similar to that of the periodic RADP ABM model (RADP = 22.6 min). Furthermore, when comparing the oscillatory behaviour shown by Shankaran and colleagues, the ABM model matches several features in the pMAPK response. Both Shankaran’s data and the ABM model show similar “turn off” dynamics for all the oscillatory waves and the maintenance of the oscillatory behaviour past the first response trigger. The ABM (with 4.5 ≤ RADP ≤ 7.5 min) and some of the oscillatory behaviour in Shankaran’s paper demonstrated graded responses while continuing to oscillate until the levels of pMAPK were close to E_max_. We also noted similarities at the phase between the turn-on and turn-off phase in the oscillatory waves. Both the ABM (when 6 ≤ RADP ≤ 23 min) and some of the *in vitro* data at the initial response show some fluctuations in pMAPK levels before the “turn off” phase. In our model, we observed that this was due to a second wave of MAPKK activation which were either dormant or not in close proximity to bind to MAPK during the initial wave of activation (Fig 5F and 5H). However, the small number of available pMAPKK agents and their lengthy RADP hindered further activation of the recently available MAPKs.

The pMAPK dynamics seen in models including cluster assembly and disassembly were also similar to the results obtained with compartmentalised MAPK signalling at the endosome (S3 Fig). Lefkowitz had shown that a typical response of MAPK involving the endosome and G protein-coupled receptors (GPCRs) are divided into two phases; a GPCR- and β-arrestin-dependent phases. The GPCR-dependent phase was characterised by a rapid initial MAPK activation followed by a rapid “turn-off” phase. In contrast, the second phase is endosomal and β-arrestin-dependent and is characterised by slow activation and deactivation phases [40]. Activation dynamics of ERK (a target of the GPCR induced signalling) incorporated both the GPCR- and β-arrestin-dependent responses. In Fig 7 and S3 Fig, the ABM produced two phase MAPK activation response (a rapid activation at the initial phase followed by a slow deactivation phase). The deactivation phase was capable of lowering the levels of activated MAPK. These characteristics produced by the ABM are similar to the endosomal MAPK activation dynamics demonstrated *in vitro* by Lekowitz. This might suggest that the formation of signalosome clusters at subcellular compartments could generate signals comparable to those triggered at membrane clusters.

### A Multi-Compartment Model Combined with Multiple MAPKK Re-Activation Delay Periods (RADPs) Reveals that the Rate and Level of pMAPK Formation is Influenced by MAPKK RADPs

Cells reside in a dynamically changing environment. A highly studied example of such dynamic environments is development and/or differentiation. During somatogenesis, cells are exposed to strong signals and potent feedback control mechanisms; both of which are periodic and oscillatory in nature [41, 42].

**Fig 8:**
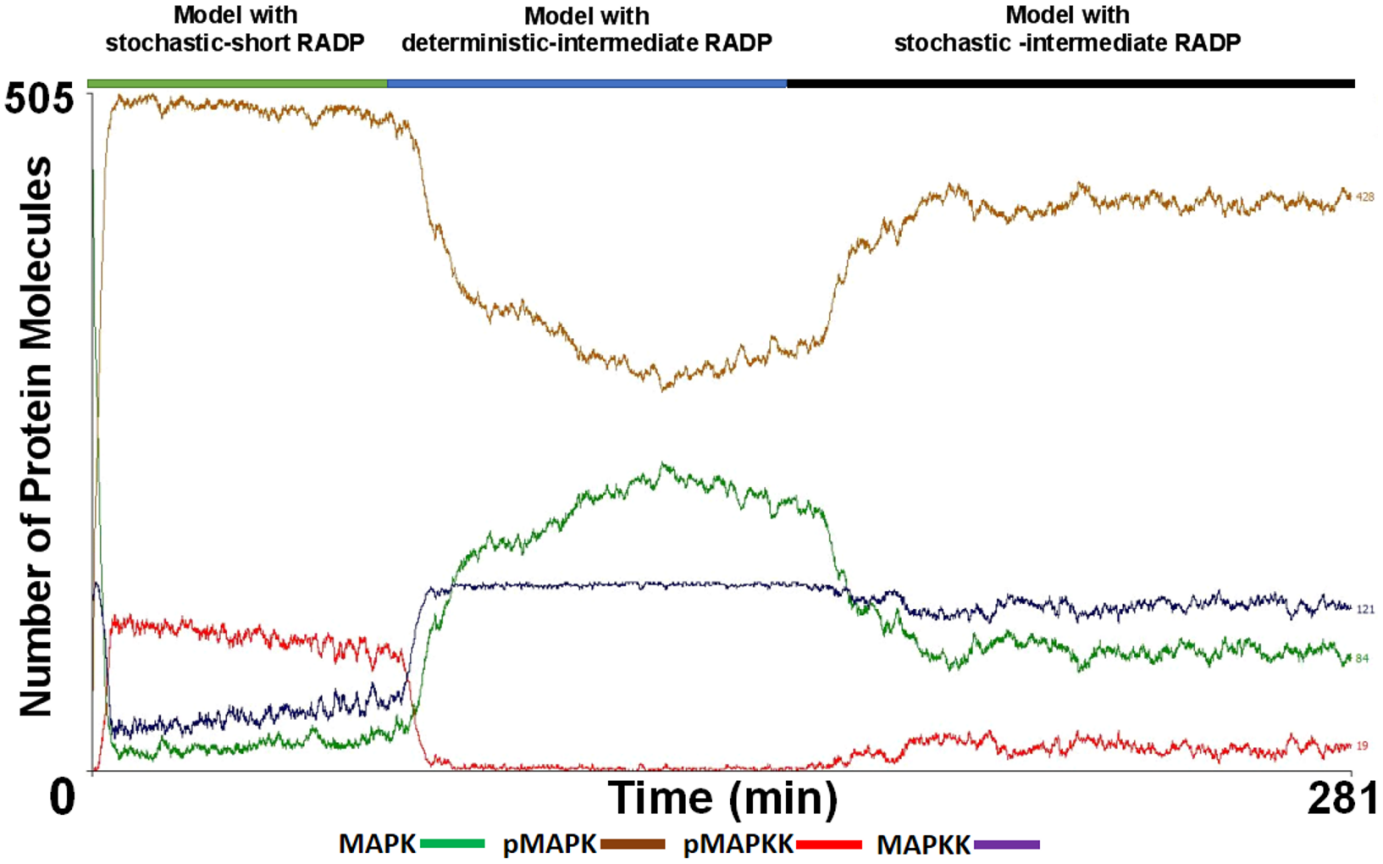
The effect of changeable input-output dynamics at the level of MAPKK on phosphorylated MAPK (pMAPK) formation characteristics in a multi-compartment system. Using the multi-compartment model, the MAPK pathway was run with different re-activation delay period (RADP) configurations to assess how switching between different MAPKK dormancy periods affect the formation of pMAPK. This was done to resemble a cellular system where a cell is initially faced with a strong, yet short activating signal, followed by the take-over of the inhibitory mechanisms, which is subsequently succeeded by a moderate and persistent activating signal. This simulation is similar to what cells are exposed to during somatogenesis. In the initial phase, a highly stochastic model of MAPKK RADP (0 ≤ RADP ≤ 90 s) was used (green solid line), once pMAPK level reached its maximum and was at equilibrium, the simulation was switched to deterministic-intermediate RADP model (RADP = 7.55 min, solid blue line). Once the level of pMAPK reached its lowest and was at equilibrium, the re-activation delay was switched to a model with stochastic-intermediate RADP (0 ≤ RADP ≤ 7.55 min; solid black line). This combination of the different modes of the MAPKK re-activation shows that once strong activation inputs of MAPKK are substantially reduced, inhibitory inputs which cause the deactivation of pMAPKK for long periods are capable of rapidly reducing the levels of pMAPK. However, they are still not capable of re-establishing the initial levels of MAPK seen at t_0_ as only 58.7% of t_0_ MAPK level was re-established. The final stage of the simulation (solid blue lines), reflects that in a multi-compartment system, even with a high stochasticity for MAPKK activation, a low number of active pMAPKK is sufficient to fundamentally increase and maintain high pMAPK levels.

The MAPK pathway is thought to be triggered during somatogenesis by fibroblast growth factor (FGF) with ERK and dual specificity phosphatase gene Dusp4 both playing a role in this process [43]. ABM was used to test system recovery and the reversibility of pMAPK levels once E_max_ had been reached by replicating the dynamic changes in external signals that have previously been reported experimentally. This was implemented by employing a combined multi-compartment model. In this model, a strong initial signal was applied which was then succeeded with a strong inhibitory response, followed by a model with a periodic activation of MAPKK. This was achieved by combining three RADP configurations and merging them into the multi-compartment ABM. Activation of the multi-compartment model was initiated with a highly stochastic-short RADP model (0 ≤ RADP < 90 s); this led to an accelerated rate of pMAPK formation and a rapid reduction in MAPK levels (Fig 8, solid green line). Once the steady state levels of pMAPK were reached, deterministic-intermediate RADP (RADP = 7.55 min) was switched on (Fig 8, solid blue line). This was to mimic a strong inhibitory signal capable of dephosphorylating and thus deactivating MAPKK. Once the lowest steady state levels of both MAPKK and pMAPK were established, the model was switched to a stochastic-intermediate RADP model (0 ≤ RADP < 7.55 min; Fig 8 with the solid dark lines). Switching to a deterministic model with an intermediate RADP (strong and sustained inhibitory feedback) led to reduced levels of pMAPK (ca. 50% of the maximum), while showing a very rapid inhibition of MAPKK (ca. 95.9%). Behaviour of the stochastic model with an intermediate RADP demonstrates that low levels of pMAPKK and a slow rate of conversion of MAPKK to pMAPKK were capable of rapidly establishing high pMAPK levels and producing an ultrasensitive activation behaviour.

## Discussion

The dynamics of the MAPK pathway has been investigated widely using *in silico* models [33]. Since the publication of Ferrell’s first model of the pathway, many more models have been reported. The majority of these papers predicted patterns and mechanisms in the pathway which explained experimental observation(s) [44]. Some of the most influential studies include the works of Levchenko who explained the contradictory experimental observations scaffold proteins have on the activation of signalling systems and the work of Ferrell *et al* and Kholodenko which explored the effect of negative and positive feedback loops on system behaviour [22, 45]. Levchenko’s model showed that scaffold concentrations in the cell are responsible for these contradictions and that scaffolds have to be within a critical concentration in order to enhance MAPK signalling [46]. Other models revealed that negative and positive feedback loops are needed for the emergence of bistability and ultrasensitivity [24, 25, 47]. Furthermore, the works of Sarma *et al* had predicted that based on the architecture and feedback mechanisms of the MAPK pathway, the formation of phosphorylated species of ERK should exhibit oscillatory behaviour [23]. This was prediction was confirmed experimentally only recently [21, 48].

The most commonly used approach to model the MAPK pathway is to use ODEs to describe the pathway and the reactions, which lead to the formation of the phosphorylated species at the three tiers. In our study, we used an agent based model (ABM) approach as it enabled us to investigate system behaviour whilst also gaining an insight into the faith of individual proteins, the physical interaction between them and their environment in addition to the spatial parameters of the model. The latter is something unfortunately ODEs cannot address [49–51]. In this ABM approach, a generalised model of the MAPK pathway had been used. This was done for a few reasons. First, a generalised model would be able to investigate effects, which could then be applied to specific MAPK pathways and thus more transferable and testable in a number of experimental settings. Secondly, there is limited experimental information regarding to MAPK compartment numbers, the physical interactions occurring in them or the number of individual MAPKs in each compartment and their impact on signalling. Furthermore, a generalised MAPK pathway model integrates, to some extent, the influence of other pathways into the MAPK signalling network (such as feedback loops).

Our ABM, as shown in Fig 2A is composed of the second and third tiers of the MAPK pathways. It allows MAPKK to become activated by an upstream stimulus, which in biological systems is transmitted via the first tier (MAPKKK) of the cascade. The model primarily relied on the physical interactions and binding properties between MAPKK and MAPK and was used to study the impact of compartmentalisation and the inputs into the cascade (both inhibitory and activating inputs) at the MAPKK level. The model implemented competitive inhibition and sequestration interactions between MAPKK and MAPK, as described previously in several experimental studies [52–54]. This was achieved by the change of state of pMAPKK to a dormant state once it activated MAPK. It has previously been suggested that competitive inhibition and sequestration-based-feedback between pMAPKK and MAPK play a role in the dynamics of MAPK pathway and they are capable of producing ultrasensitivity and bistability in the system and thus influence the cellular outcome [55].

The initial design of the model employed a system that contained very low competitive cooperative inhibition and sequestration of the MAPKK. Similar to the majority of previously published MAPK models, it involved two-compartments with the interacting species moving around the “cytoplasm” in Brownian motion. However, this implementation of the ABM only produced a graded activation response for the pathway. Increasing diffusion parameters in ODE models has previously been shown to be responsible for decreasing reaction orders and thus MAPK activation following Michaelis–Menten kinetics [56, 57]. Once diffusion parameters are reduced, such as when seen in the presence of scaffold proteins, the reaction order had increased, increasing the rate of phosphorylation and led to ultrasensitive MAPK response [46, 58]. In cells, if phosphorylated species were to rely only on diffusion to propagate the signal downstream, an increased probability of phosphatase action would lead to the reduction of the reaction rate [50, 59, 60]. However, *in silico* models show that this could be overcome by spatially restricting phosphatases and kinases in the cell and consequently, the formation of local pools leading to the localisation of the signal [61]. In the ABM, relying solely on Brownian motion lowers the probability of direct interactions between MAPKK and MAPK species, and even in the absence of phosphatases or inhibitory enzymes, pathway activation does not lead to strong ultrasensitivity. This behaviour matches well with findings reported in the ODE-based and experimental studies discussed above.

The introduction of multi-compartmentalisation in previous studies led to ultrasensitive response as well as oscillatory behaviour in the system. Legewie et al, Ortega et al and Qiao et al demonstrate that variations of parameters have an effect on the final response of the system and their variation might be responsible for distinct outputs [53, 56, 62]. They also show that only few of these parameters are capable of generating bistability and/or oscillation. However, they highlight that all of this hinges on phosphorylation cycles, and that the main contributors to these effects are the small numbers of regulatory molecules in the pathway. Our ABM shows that varying the input parameters at the level of MAPKK is capable of producing two distinct responses to a signal; nonetheless, it also demonstrates that compartmentalisation as well as mode of the output at the level of the MAPKK could play an important role for the generation of ultrasensitivity and oscillation.

In the ABM that included multi-compartments, a prominent ultrasensitive response emerged. This occurred in the presence of competitive inhibition and even when sequestration interaction between the pMAPKK and MAPK species was high (when RADPs > 15 min, Fig 5G). In a model where the RADP was stochastic, the rate of the phosphorylated MAPK species formation and thus the magnitude of the MAPK response had only significantly decreased when RADP ≥ 8 min (S2A Fig). This is interesting as it was shown experimentally that pMAPK magnitude play a role in the specificity and fidelity of the MAPK pathway [38, 55]. This also implies that compartmentalisation could play a role in allowing for fidelity to a response regardless of the strength of input at the level of the MAPKK.

Oscillation in the MAPK pathway is strongly linked to negative feedback loops; though there is also a realisation that balance between positive and negative feedback is fundamental as these are being shown both *in vitro* and *in silico* [21, 24, 63]. Several modelling approaches showed that the outcome of feedback loops differ depending on the mode of the feedback applied. Moreover, the position of these feedback loops within the cascade’s three tier architecture influence the output and hence the behaviour of the cascade [23–25]. In the model presented here, balance between negative and positive feedback loops were taken into account by relying on the final output of inhibitory *versus* activating inputs from feedback loops at the level of MAPKK. This was implemented by the introduction of the re-activation delay periods (RADP). The model shows that when RADPs were deterministic (*i*.*e*. periodic), oscillatory behaviour emerged; in-line with previous observations which illustrate that once strong negative feedback loops were applied, oscillation was generated. In the ABM, the frequency of the oscillation and the amplitude were both influenced by RADPs. This is interesting as it was shown experimentally that frequency and amplitude of phosphorylated ERK influence the expression of specific genes such as c-Fos [20, 64]. It has also been proposed that oscillation might be a mechanism by which MAPK signalling is restricted to the cytoplasm as the frequency and amplitude would affect the MAPK targets in the cytoplasm [16]. The appearance of oscillation within the multi-compartment model strengthens this argument. Compartmentalisation and the periodicity of input at the MAPKK level could act as a filter and/or modulator for localised responses. Compartmentalised MAPK targets would be directly available to interact with phosphorylated species of MAPK, however, if there are multiple targets, their ability to react differentially to the same input signal (i.e. de-coding capabilities) would specify a hierarchy of interactions within the compartment and therefore control the development of the specific response.

Our results presented above demonstrated that with long periodic RADPs, oscillation becomes sustained; this is consistent with previous observations that sustained oscillation appear in models which also exhibit ultrasensitivity and strong negative feedback inhibition. However, the ABM also shows that periodic MAPKK activation and multi-compartmentalisation are essential for sustained oscillation to appear. Previously, oscillation was described as a random process, which could emerge in the absence of regulatory mechanisms, yet the ABM demonstrated that altering the periodicity of RADP at the level of MAPKK in a multi-compartment model is integral for oscillation to appear. This was confirmed when the ABM was converted to a two-compartment model and the effects of RADPs were re-tested (Fig 6). In addition, oscillation emerged in a relatively simple model suggesting that for oscillation to appear specific parameters need to be met [56, 57]. This might be plausible considering that oscillation does not appear experimentally when a population of cells is monitored, yet, it can be observed at the level of individual cells. This could suggest that the conditions required for oscillation are more easily met at the single-cell level, compared to cell populations [21, 48].

Signalosome clustering at the plasma membrane has been reported previously [37, 65] and was shown to contribute to MAPK cascade’s specificity and efficacy [66]. Chiu *et al* demonstrated that Ras-nanoclusters could also be formed in cytoplasmic membranes [67]. However, Tian *et al* and others proposed that plasma membrane Ras nanoclusters are essential for MAPK activation and are major contributors to the rapid activation observed at the initial phase of global MAPK activation response [39, 68, 69]. In addition, Inder *et al* suggested that endoplasmic reticulum and Golgi Ras nanoclusters play a role in the differentiation of the incoming signal and thus determining the response output [70]. On the other hand, the off phase of MAPK activation is attributed to the disassembly of signalosome clusters, followed by diffusion into the cytoplasm [60, 68, 71]. The ABM described here (Fig 7) demonstrates, indeed, clustering of MAPKK and MAPK is responsible for the initial, robust activation of MAPK and that the disassembly of these components is responsible for the characteristics of the off phase.

Considering that compartmentalisation is a fundamental property of the cell and components of the signalosome are found inside of the compartments, the ABM reported here strengthens the argument that plasma membrane clustering might not be the sole contributor to signal efficiency and specificity and that compartments within the cytoplasm may be capable of mediating similar effects. Additionally, if both plasma membrane and cytoplasmic membrane clusters contribute to MAPK activation, their combined effects should be synergistic. This might be a valid postulation considering that Ras clusters at the ER and Golgi were experimentally shown to be triggered by Raf1, which could be triggered at the plasma membrane [70]. Such combination of plasma membrane-originated and cytoplasm-originated activation of MAPK might also be a source for generating oscillation as cytoplasmic clusters would re-trigger MAPK activation. Alternatively, if both plasma and cytosolic clusters were simultaneously activated as reported in some MAPK systems [72, 73], in order to generate the usually observed global MAPK response, strong negative feedback loops, insulation and isolation mechanisms should be present at amplification points within the MAPK system.

The ABM also showed similarities with other *in silico* models. As mentioned previously, *in silico* models in general and ODEs in particular have been very insightful in explaining and improving our understanding of signal transduction and signal processing. However, ODEs are limited in modelling spatial constraints, and with them it is challenging to model individual protein-protein interactions in multi-protein complexes. For partial differential equations (PDEs) the limitation lies in the complexity of writing several mathematical expressions and equations for every compartment and the corresponding equations, which allow those to change over time. We choose to use the ABM as it overcomes these limitations and we have validated our approach with previously published data obtained from ODEs and PDEs. Furthermore, the ABM with periodic and long RADP shared similarities with the oscillatory pattern of pMAPK vs. MAPK in a models published by Kholodenko et al, employing negative feedback and competitive inhibition [22]; the latter is also an important characteristics of the ABM. The periodicity of oscillations was also very similar between the two models. The graded response, combined with oscillation seen at the initial activation phase generated by the ABM with RADP = 4.5 min (Fig 5D); is similar to the dynamics of MAPK activation as demonstrated by a Zhao *et al* in a model of the MAPK pathway using PDEs [74]. However, there were also differences between the ABM and PDE models in the time frame of achieving E_max_. Additionally, Zhao’s model had achieved a higher frequency and continuous oscillation at E_max_, while we did not see maintenance of high frequency in our ABM implementations.

The presented model contributes to a mechanistic analysis of the dual effects of spatio-temporal regulation of MAPK pathways and suggests that ultrasensitivity and oscillation emerge in the pathway as a product of coupled spatiotemporal modulation and that multi-compartmentalisation might be an important and integral factor for these behaviours to occur.

## Concluding Remarks

In this study, we investigated the dynamics of MAPK pathway activation in both a two compartment- and a multi compartment-model. We showed that compartmentalisation has an important effect on three aspects of pathway activation. The first is the magnitude of response once the pathway is turned on, the rate by which the system reaches equilibrium and recovers from the initial activation and finally how oscillation at the level of MAPK/pMAPK could arise by periodic activation of MAPKK coupled with compartmentalisation of pathway components. Our models also demonstrated that in order to achieve levels of MAPK close to those at t_0_, the MAPKK should be under moderate to high inhibitory feedback regulation. Additionally, the dynamics of MAPK activation obtained from the ABM model share many parallels with observed MAPK dynamics both *in vitro* and *in silico*.

## Methods

For the construction of the model, the agents were modelled as stream X-machines. There are four components fundamental to X-machines, these are inputs, outputs, state memory and functions. Inputs and state memory get processed by the X-machine using finite-functions. Subsequently, the X-machine transition occurs. Transition functions map to a new X-machine state and to an output. As a result, a new X-machine state is achieved with new sets of functions. These new functions dictate the input accepted by the X-machine, the states the X-machine could transform to, the functions and outputs associated with the X-machine.

To implement this, descriptions of the agents were written in Extensible Markup Language (XML) while for the execution of the model the source code was written with C language. To create the agents and run the model, Flexible Large-scale Agent Modelling Environment (FLAME) framework was used with iterated time-steps [75–77].

Iterated time-steps were converted to minutes by first analysing MAPK activation patterns reported in the literature from *in vitro* data. The times taken to generate E_*max*_ response of activated MAPK species were calculated and the mean and mode values were determined from all the graphs (Fig 2). The average time was 8.98 (min) ± 5.08 (mean ± SD) and the median was 7.73 (min). We opted for the median as statistical analysis shown that the data was not normally distributed. This time value was used to convert time-steps taken to generate the maximum response in the ABM model into minutes.

### Initial Conditions and Basic Model Structure

#### Agent numbers

The number of the different components of the MAPK cascade (MAPKK and MAPK) in the model at t = 0 was determined from protein concentrations described by Huang et al and Chickarmane V et al [45, 78]. These concentrations were converted to moles by adapting the average number of mean corpuselar volume of red blood cells (≈90 femtoliter) as the volume these proteins were present in (as they are mainly cytoplasmic). Moles were then converted into number of protein molecules using Avogadro's number. See Table 1 below.

**Table 1:** 

Agent name	Number of protein agent molecules in the two compartment model	Number of protein agent molecules in the multi-compartment model
MAPKK (MAPKK)	0	100
phosph-MAPKK (pMAPKK)	500	20
MAPK (MAPK)	500	0
phosph-MAPK (pMAPK)	250	500
ExR (active)	180	180
ExR (dormant)	180	180

This is where MAPKK activates MAPK leading to the formation of pMAPK, which translocates to the nucleus. Once translocated to the nucleus MAPK could interact with active exporting receptors (ExR) in order to translocate out of the nucleus. This scheme is represented in Fig 2A.

### Rules Governing Agents’ Behaviour

Simple rules were assigned to the agents in both models. These rules specified the agents’ movement and the manner with which they interacted with their interaction partners.

### Agents’ Description

Both models contained the same agents were. The agents were separated into cytoplasmic and nuclear species:

### Cytoplasmic agents

#### MAPKK (MKK)

MAPKK agent is found in two states, pMAPKK and MAPKK. pMAPKK only interacts with MAPK agent. It reads locations messages of the different MAPK agents, this allows it to determine the closest MAPK available for binding. pMAPKK sends location messages and binding status messages to close by MAPK agents. Once confirmation of binding availability is established between MAPK and MAPKK, binding occurs. This leads to the change in pMAPKK state to the dormant MAPKK (MAPKK). MAPKK reverts back into pMAPKK after a lag phase (the re-activation delay period, RADP). A RADP value assigned to individual MAPKKs and it becomes updated once MAPKK returns back to pMAPKK. RADP value was updated either deterministically or stochastically (Fig 2E). For the deterministic update, the value (for every MAPKK agent) was identical and it was the upper limit chosen for any particular simulation. For the stochastic updating, RADP was set (for individual MAPKK agent) randomly at a value between 0 and the chosen RADP upper limit. MAPKK moves by Brownian motion. In the two compartments model, this movement is restricted to the cytoplasm, where MAPKK deflects off the plasma membrane and the nuclear membrane. While in the multi-compartment model this movement is restricted to the individual compartment boundaries.

#### MAPK (MAPK)

MAPK interacts with a number of agents in the model. It sends messages of its location and binding availability which are read by these agents. Once the binding availability become confirmed MAPK interact with the given agent. MAPK interacts with MAPKK in the cytoplasm, and with ExR at the internal surface of the nuclear membrane. MAPK interacts with pMAPKK leads to MAPK activation, change of status to pMAPK and the translocation to the nucleus. Once in the nucleus, pMAPK also interacts with ExR, this interaction leads to the translocation of pMAPK back to the cytoplasm and/or its specific compartment in them multi-compartment system; and the reformation of MAPK.

MAPK move by Brownian motion. However, the movements of the different states are dissimilar. MAPK is restricted to move in the cytoplasm or within the boundary of its specific compartment only. On the other hand, pMAPK are restricted to move within the cytoplasm.

### Nuclear agents

#### Exporting Receptor (ExR)

There are two states for ExR, an active (ExR) and inactive (dExR). These two states are interchangeable. Exporting receptors are shifting between active and inactive states and *vice versa*. Both receptors move around by Brownian motion, however within the nuclear membrane. dExRs shift back to ExR after a lag phase (dormancy period). ExR interacts with pMAPK. The ExR receives location messages from pMAPK, close ExR respond by sending messages to closest pMAPK confirming the availability to bind. Once ExR binds to pMAPK it changes state to dExR and triggers pMAPK translocation out of the nucleus and status change to MAPK.

#### ABM codes

The complete code of the ABMs presented in this study has been uploaded on GitHub: https://github.com/MadinaJNR/Multi-Compartment-ABM-source-code-in-C-programming-language-

## Supporting Information

S1 Fig RADP stochasticity modulation and its effects on MAPK activation dynamics. Stochastic RADP configurations were tested by varying the RADP ranges in the multi-compartment ABM. (A) RADP value was set to be generated within the following range 3.77 ≤ RADP < 4.55 min. At the initial activation phase minor oscillatory responses emerge. (B) Illustrates the RADP configuration when the range was set at 4.15 ≤ RADP < 4.55 min, whereby at the initial MAPK activation phase sharper miniature oscillatory activity appears. (C) Demonstrates a RADP configuration when the range was set at 4.38 ≤ RADP < 4.55 min, there the miniature oscillatory activity become more visible. This last RADP configuration is the least stochastic due to its limited range for RADP re-setting value, thus the MAPK activation behaviour is analogous to the deterministic configuration where RADP = 4.55 min.
(TIF)

S2 Fig Effects of stochastic RADP on pMAPK and MAPKK activation dynamics. (A) The pMAPK levels with each RADP configuration were examined, when RADP was less than 7.55 min, there was no significant difference between pMAPK levels compared to the control run. However, when RADP value was ≤ 7.55 min, the level of pMAPK started to become significantly lower compared to the control run, with 0 ≤ RADP ≤ 22.65 min, demonstrating a substantial significance. (B) Conversely, the time to achieve Emax appeared to be significantly different when RADP was less than 22.63 min. (C) When the time to achieve EC50 was considered, only 0 ≤ RADP ≤ 22.63 min configuration illustrated a significant difference compared to control run. (D) When the effect of the RADP configuration was examined in relation to MAPKK, increasing RADP caused a significant reduction in the level of active MAPKK. (E) The increasing RADP value prompted an increase in the time to achieve Emax when RADP configuration was RADP ≤ 22.65 min. (F) This was also reflected with significant increase in the time to achieve EC50, yet, when RADP range was within 22.63 min the time to achieve EC50 was significantly. This is due to the significantly small magnitude of MAPKK generated in comparison to the contro. N = 3, one way ANOVA test was conducted to demonstrate significance with *, ** and *** corresponding to p < 0.05, p < 0.001 and p < 0.0001 respectively.
(TIF)

S3 Fig MAPK activation dynamics; AMB vs. experimental data. Relative pMAPK levels were compared between experimental data, reported by Lefkowitz RJ *et al*. [40] vs. our ABM. Multiple t-tests were performed with Holm-Sidak corrections for multiple comparisons. No significant differences were observed.
(TIFF)

S1 File Detailed description of agent memory, messages and functions. (DOCX)

S2 File List of references used for calibration of MAPK activation in the ABM. (DOCX)

S3 File Examples of RADP values generated in the stochastic ABMs. (XLSX)
