# Supplementary figures and images for: Multi-Compartmentalisation in the MAPK Signalling Pathway Contributes to the Emergence of Oscillatory Behaviour and to Ultrasensitivity
Source: PLoS One. 2016 May 31;11(5):e0156139. doi: 10.1371/journal.pone.0156139 (PMC4887093; doi:10.1371/journal.pone.0156139)

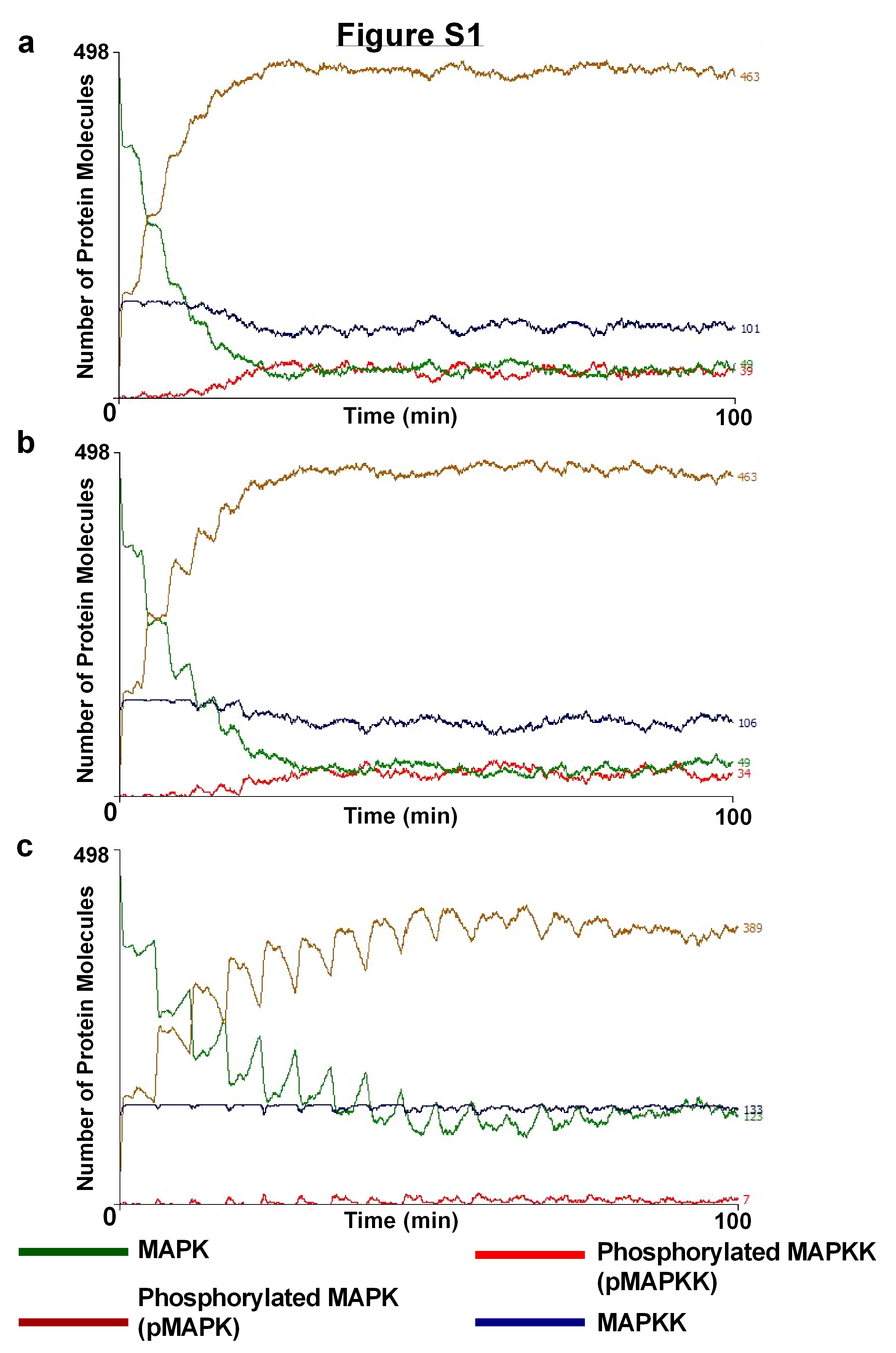

Supplement: S1 Fig — Stochastic RADP configurations were tested by varying the RADP ranges in the multi-compartment ABM. (A) RADP value was set to be generated within the following range 3.77 ≤ RADP < 4.55 min. At the initial activation phase minor oscillatory responses emerge. (B) Illustrates the RADP configuration when the range was set at 4.15 ≤ RADP < 4.55 min, whereby at the initial MAPK activation phase sharper miniature oscillatory activity appears. (C) Demonstrates a RADP configuration when the range was set at 4.38 ≤ RADP < 4.55 min, there the miniature oscillatory activity become more visible. This last RADP configuration is the least stochastic due to its limited range for RADP re-setting value, thus the MAPK activation behaviour is analogous to the deterministic configuration where RADP = 4.55 min. (TIF) [file pone.0156139.s001.tif]

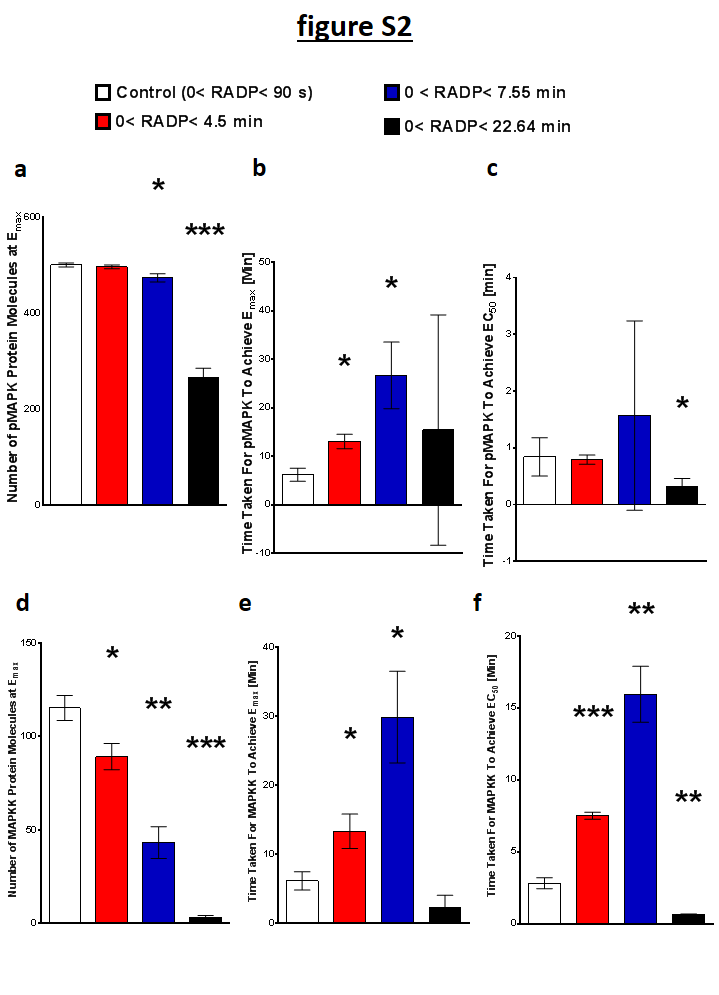

Supplement: S2 Fig — (A) The pMAPK levels with each RADP configuration were examined, when RADP was less than 7.55 min, there was no significant difference between pMAPK levels compared to the control run. However, when RADP value was ≤ 7.55 min, the level of pMAPK started to become significantly lower compared to the control run, with 0 ≤ RADP ≤ 22.65 min, demonstrating a substantial significance. (B) Conversely, the time to achieve Emax appeared to be significantly different when RADP was less than 22.63 min. (C) When the time to achieve EC50 was considered, only 0 ≤ RADP ≤ 22.63 min configuration illustrated a significant difference compared to control run. (D) When the effect of the RADP configuration was examined in relation to MAPKK, increasing RADP caused a significant reduction in the level of active MAPKK. (E) The increasing RADP value prompted an increase in the time to achieve Emax when RADP configuration was RADP ≤ 22.65 min. (F) This was also reflected with significant increase in the time to achieve EC50, yet, when RADP range was within 22.63 min the time to achieve EC50 was significantly. This is due to the significantly small magnitude of MAPKK generated in comparison to the contro. N = 3, one way ANOVA test was conducted to demonstrate significance with *, ** and *** corresponding to p < 0.05, p < 0.001 and p < 0.0001 respectively. (TIF) [file pone.0156139.s002.tif]

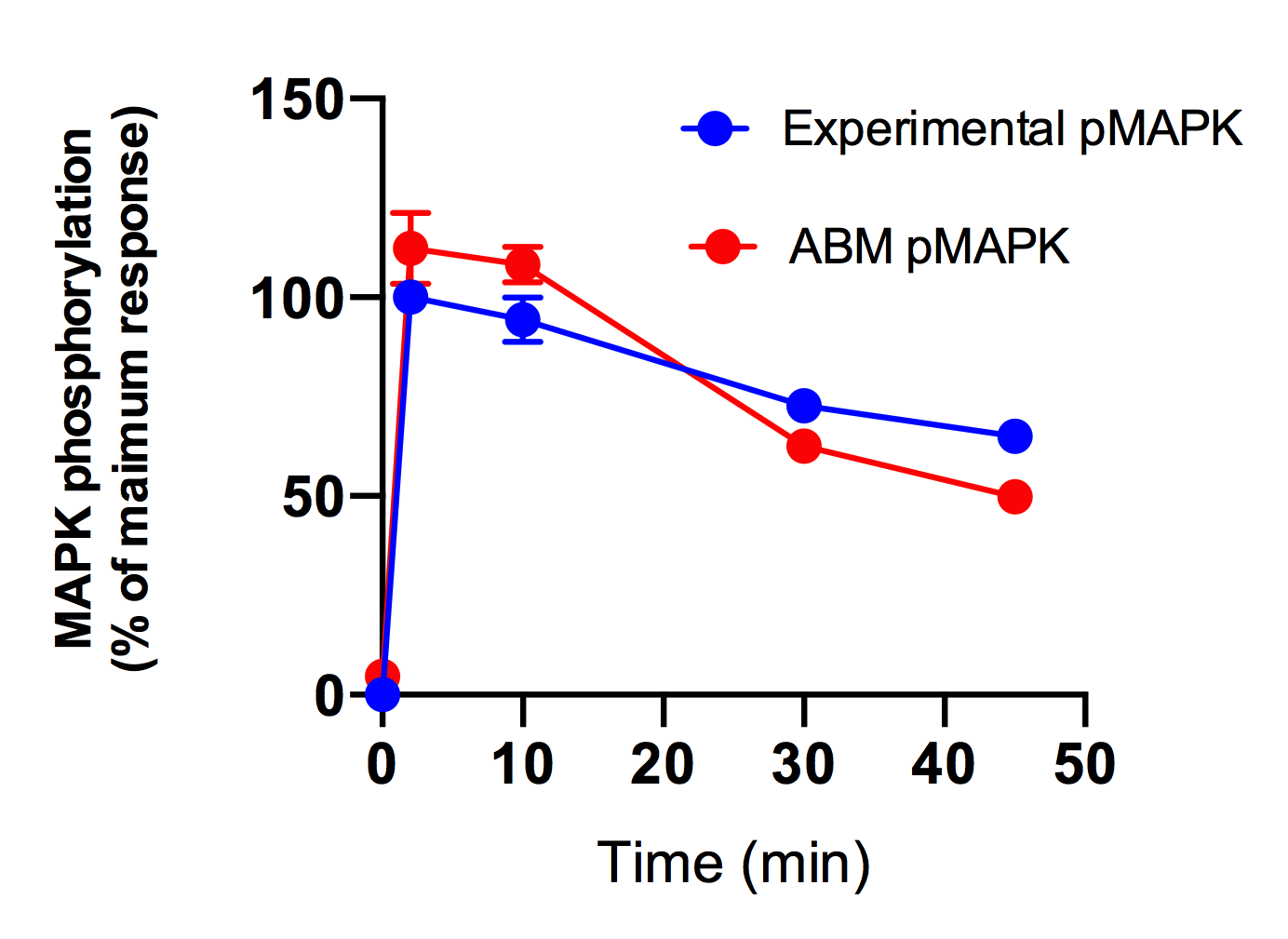

Supplement: S3 Fig — Relative pMAPK levels were compared between experimental data, reported by Lefkowitz RJ et al. [40] vs. our ABM. Multiple t-tests were performed with Holm-Sidak corrections for multiple comparisons. No significant differences were observed. (TIFF) [file pone.0156139.s003.tiff]
