# Supplementary material for: Multi-Compartmentalisation in the MAPK Signalling Pathway Contributes to the Emergence of Oscillatory Behaviour and to Ultrasensitivity
Source: PLoS One. 2016 May 31;11(5):e0156139. doi: 10.1371/journal.pone.0156139 (PMC4887093; doi:10.1371/journal.pone.0156139)
Supplement: S1 File — (DOCX) [file pone.0156139.s004.docx]

**Supporting Information**

Text S1.

Formal description of an agent

The tables below were constructed in order to clearly describe the agents’ states, memory functions and the interaction rules using the messages send and received.

**Agent Memory**

| **MAPKK** | | |
| --- | --- | --- |
| **Name** | **Type** | **Description** |
| id | int | ID for agent |
| comptag | double | Tag to identify which compartment MKK resides in |
| postheta | double | Agent position in the spherical coordinate system (θ is the angle rotating about the y-axis, where y coordinates is expressed as the function SinθSinφ |
| posphi | double | Agent position in the spherical coordinate system (φ is the angle rotating about the Z-axis, where Z coordinate is expressed as the function Cosφ |
| posr | double | the position of the agent expressed in radians |
| posx | double | the position of the agent on the X-axis |
| posy | double | the position of the agent on the Y-axis |
| posz | double | the position of the agent on the Z-axis |
| movetheta | double | Movement angle in the spherical coordinate system (θ is the angle between z-axis and r) |
| movephi | double | Movement angle in the spherical coordinate system (φ is the angle between x-axis and r.cosθ) |
| mover | double | The displacement of the agent in the spherical coordinate system (~r or p) |
| state | int | MAPKK state (e.g. phosphor-MAPKK [pMAPKK] or MAPKK) |
| RADP | int | The re-activation delay periods which defines the time period which MAPKK stays dormant before it reverts back to MKK |
| boundindex | int | Determines the bound state of MKK to MAPK |
| iradius | double | which defines the number of iterations a protein state lasts before it changes to or reverts back to another state |

| **MAPK** | | |
| --- | --- | --- |
| **Name** | **Type** | **Description** |
| id | int | ID for agent |
| comptag | double | Tag to identify which compartment MAPK resides in |
| postheta | double | Agent position in the spherical coordinate system (θ is the angle rotating about the y-axis, where y coordinates is expressed as the function SinθSinφ |
| posphi | double | Agent position in the spherical coordinate system (φ is the angle rotating about the Z-axis, where Z coordinate is expressed as the function Cosφ |
| posr | double | the position of the agent expressed in radians |
| posx | double | the position of the agent on the X-axis |
| posy | double | the position of the agent on the Y-axis |
| posz | double | the position of the agent on the Z-axis |
| movetheta | double | Movement angle in the spherical coordinate system (θ is the angle between z-axis and r) |
| movephi | double | Movement angle in the spherical coordinate system (φ is the angle between x-axis and r.cosθ) |
| mover | double | The displacement of the agent in the spherical coordinate system (~r or p) |
| state | int | MAPK state (e.g. MAPK or phosphor-MAPK [pMAPK]) |
| iradius | double | which defines the number of iterations a protein state lasts before it changes to or reverts back to another state |

| **ExR** | | |
| --- | --- | --- |
| **Name** | **Type** | **Description** |
| id | int | ID for agent |
| postheta | double | Agent position in the spherical coordinate system (θ is the angle rotating about the y-axis, where y coordinates is expressed as the function SinθSinφ |
| posphi | double | Agent position in the spherical coordinate system (φ is the angle rotating about the Z-axis, where Z coordinate is expressed as the function Cosφ |
| posr | double | the position of the agent expressed in radians |
| posx | double | the position of the agent on the X-axis |
| posy | double | the position of the agent on the Y-axis |
| posz | double | the position of the agent on the Z-axis |
| movetheta | double | Movement angle in the spherical coordinate system (θ is the angle between z-axis and r) |
| movephi | double | Movement angle in the spherical coordinate system (φ is the angle between x-axis and r.cosθ) |
| mover | double | The displacement of the agent in the spherical coordinate system (~r or p) |
| state | int | ExR state (e.g. ExR or dExR) |
| recdelay | int | The re-activation delay periods which defines the time period which ExR stays active before it changes to dExR and the time period dExR state exist before it it reverts back to ExR |
| boundindex | int | Determines the bound state of ExR to phospho-MAPK |
| iradius | double | which defines the number of iterations a protein state lasts before it changes to or reverts back to another state |

**Messages:**

| MAPKK MESSAGES | | |
| --- | --- | --- |
| **Message Name** | **MKKlocation** | |
| Description | Details the location of MKK in the 3D coordinates and its state | |
| Elements | | |
| Name | Type | Description |
| Id | int | id of the agent (i.e. MAPKK) |
| X | double | x coordinates |
| Y | double | y coordinates |
| Z | double | z coordinates |
| State | int | agent state |
| Range | double | the agent's message range |
| **Message Name** | **MKKnewbond** | |
| Description | Message to signifies the formation of a bond between pMAPKK and MAPK | |
| Elements | | |
| Name | Type | Description |
| idfrom | int | The id of the agent which released the message (i.e. pMAPKK) |
| statefrom | int | The stare of the agent which released the message (i.e. pMAPKK) |
| Idto | int | The id of the agent the message is targeted to (i.e. MAPK ) |
| bindunbind | int | Whether to bind or not |
| distance | double | Distance between agents |
| range | double | The agent's message range |
| X | double | x coordinates |
| Y | double | y coordinates |
| Z | double | z coordinates |

| **APK MESSAGES** | | |
| --- | --- | --- |
| **Message Name** | **MKlocation** | |
| Description | Check location of agent | |
| Elements | | |
| Name | Type | Description |
| Id | int | id of the agent (i.e. MAPK ) |
| X | double | x coordinates |
| Y | double | y coordinates |
| Z | double | z coordinates |
| State | int | agent state |
| Range | double | the agent's message range |
| **Message Name** | **MKfinalbond** | |
| Description | Confirmation for the establishment of a bond between MAPK and either MKK or ExR | |
| Elements | | |
| Name | Type | Description |
| idfrom | int | The id of the agent which released the message (i.e. MAPK ) |
| statefrom | int | The stare of the agent which released the message (i.e. MAPK ) |
| Idto | int | The id of the agent the message is targeted to (i.e. MAPK K or ExR) |
| bindunbind | int | Whether to bind or not |
| distance | double | Distance between agents |
| range | double | The agent's message range |
| x | double | x coordinates |
| y | double | y coordinates |
| z | double | z coordinates |

| xR MESSAGES | | |
| --- | --- | --- |
| **Message Name** | **ExRlocation** | |
| Description | Check location of agent | |
| Elements | | |
| Name | Type | Description |
| Id | int | id of the agent (i.e. ExR) |
| X | double | x coordinates |
| Y | double | y coordinates |
| Z | double | z coordinates |
| State | int | agent state |
| Range | double | the agent's message range |
| **Message Name** | **ExRnewbond** | |
| Description | Message to signifies the formation of a bond between ExR and pMAPK | |
| Elements | | |
| Name | Type | Description |
| idfrom | int | The id of the agent which released the message (i.e. ExR) |
| statefrom | int | The stare of the agent which released the message (i.e. ExR) |
| Idto | int | The id of the agent the message is targeted to (i.e. MAPK ) |
| bindunbind | int | Whether to bind or not |
| distance | double | Distance between agents |
| range | double | The agent's message range |
| X | double | x coordinates |
| Y | double | y coordinates |
| Z | double | z coordinates |

**Functions**

| **MAPKK FUNCTIONS** | | |
| --- | --- | --- |
| **Function Name** | **MKK_outputdata** | |
| **Description** | Outputs location message | |
| **Current state** | 0 | |
| **Next state** | 1 | |
| **Flow (**n/a**)** | | |
| **Inputs** | | |
| n/a | | |
| **Outputs** | | |
| **Message name** | **To agent** | |
| MKKLocation | MAPK agents | |
| **Function Name** | **MKK_inputdata** | |
| **Description** | Read all messages from other agents | |
| **Current state** | 1 | |
| **Next state** | 2 | |
| **Flow (n/a)** | | |
| **Inputs** | | |
| **Message name** | **Filter/Operation** | **From agent** |
| MAPK location | n/a | MAPK agents |
| **Outputs** | | |
| **Message name** | **To agent** | |
| MKKnewbond | MAPK agents | |
| **Function Name** | **MKK_move** | |
| **Description** | Controls protein movement and checks binding status of MAPKK | |
| **Current state** |  | |
| **Next state** | 3 | |
| **Flow (**n/a) | | |
| **Inputs** | | |
| **Message name** | **Filter/Operation** | **From agent** |
| MKfinalbond | n/a | MAPK agents |
| **Outputs** | | |
| n/a | | |

| MAPK FUNCTIONS | | |
| --- | --- | --- |
| **Function Name** | **MK_outputdata** | |
| **Description** | Outputs location message | |
| **Current state** | 0 | |
| **Next state** | 1 | |
| **Flow (**n/a) | | |
| **Inputs** | | |
| n/a | | |
| **Outputs** | | |
| **Message name** | **To agent** | |
| MKLocation | MAPKK agents | |
| **Function Name** | **MK_inputdata** | |
| **Description** | Read all messages from other agents | |
| **Current state** | 1 | |
| **Next state** | 2 | |
| **Flow (**n/a) | | |
| **Inputs** | | |
| **Message name** | **Filter/Operation** | **From agent** |
| MKKlocation | n/a | MAPKK agents |
| ExRlocation | n/a | ExR agents |
| **Outputs** | | |
| n/a | | |
| **Function Name** | **MK_checkbondtries** | |
| **Description** | Check if there were any binding attempts between MAPK and pMAPKK MK and ExR | |
| **Current state** | 2 | |
| **Next state** | 3 | |
| **Flow (**n/a) | | |
| **Inputs** | | |
| **Message name** | **Filter/Operation** | **From agent** |
| MKKnewbond | n/a | MAPKK agents |
| ExRnewbond | n/a | ExR agents |
| **Outputs** | | |
| **Message name** | **To agent** | |
| MK finalbond | All agents | |
| **Function Name** | **MK_move** | |
| **Description** | Controls protein movement and checks binding status of MKK | |
| **Current state** | 3 | |
| **Next state** | 4 | |
| **Flow (**n/a) | | |
| **Inputs** | | |
| n/a | | |
| **Outputs** | | |
| n/a | | |

| ExR FUNCTIONS | | |
| --- | --- | --- |
| **Function Name** | **ExR_outputdata** | |
| **Description** | Output location message and check nuclear receptor timers | |
| **Current state** | 0 | |
| **Next state** | 1 | |
| **Flow (** n/a) | | |
| **Inputs** | | |
| n/a | | |
| **Outputs** | | |
| **Message name** | **To agent** | |
| ExRlocation | MAPK agents | |
| **Function Name** | **ExR_inputdata** | |
| **Description** | Read all messages from other agents | |
| **Current state** | 1 | |
| **Next state** | 2 | |
| **Flow (** n/a) | | |
| **Inputs** | | |
| **Message name** | **Filter/Operation** | **From agent** |
| MKlocation | n/a | MAPK agents |
| **Outputs** | | |
| **Message name** | **To agent** | |
| ExRnewbond | MAPK agents | |
| **Function Name** | **ExR_move** | |
| **Description** | movement and check bond messages | |
| **Current state** | 2 | |
| **Next state** | 3 | |
| **Flow (** n/a) | | |
| n/a | | |
| **Inputs** | | |
| **Message name** | **Filter/Operation** | **From agent** |
| MKfinalbond | n/a | MAPK agents |
| **Outputs** | | |
| **Message name** | **To agent** | |
| n/a | | |
