# Supplementary material for: Multi-Compartmentalisation in the MAPK Signalling Pathway Contributes to the Emergence of Oscillatory Behaviour and to Ultrasensitivity
Source: PLoS One. 2016 May 31;11(5):e0156139. doi: 10.1371/journal.pone.0156139 (PMC4887093; doi:10.1371/journal.pone.0156139)
Supplement: S2 File — (DOCX) [file pone.0156139.s005.docx]

1. Ahmed S, Grant KG, Edwards LE, Rahman A, Cirit M, Goshe MB, et al. Data-driven modeling reconciles kinetics of ERK phosphorylation, localization, and activity states. Mol Syst Biol. 2014;10:718. Epub 2014/02/04. doi: 10.1002/msb.134708. PubMed PMID: 24489118; PubMed Central PMCID: PMCPmc4023404.

2. Bashor CJ, Helman NC, Yan S, Lim WA. Using engineered scaffold interactions to reshape MAP kinase pathway signaling dynamics. Science (New York, NY). 2008;319(5869):1539-43. Epub 2008/03/15. doi: 10.1126/science.1151153. PubMed PMID: 18339942.

3. Bhalla US, Ram PT, Iyengar R. MAP Kinase Phosphatase As a Locus of Flexibility in a Mitogen-Activated Protein Kinase Signaling Network. Science (New York, NY). 2002;297(5583):1018-23. doi: 10.1126/science.1068873.

4. Borisov N, Aksamitiene E, Kiyatkin A, Legewie S, Berkhout J, Maiwald T, et al. Systems‐level interactions between insulin–EGF networks amplify mitogenic signaling. Molecular Systems Biology. 2009;5(1). doi: 10.1038/msb.2009.19.

5. Brightman FA, Fell DA. Differential feedback regulation of the MAPK cascade underlies the quantitative differences in EGF and NGF signalling in PC12 cells. FEBS Letters. 2000;482(3):169-74. doi: http://dx.doi.org/10.1016/S0014-5793(00)02037-8.

6. Chang C-w, Poteet E, Schetz JA, Gümüş ZH, Weinstein H. Towards a quantitative representation of the cell signaling mechanisms of hallucinogens: Measurement and mathematical modeling of 5-HT1A and 5-HT2A receptor-mediated ERK1/2 activation. Neuropharmacology. 2009;56, Supplement 1:213-25. doi: http://dx.doi.org/10.1016/j.neuropharm.2008.07.049.

7. Cirit M, Haugh Jason M. Data-driven modelling of receptor tyrosine kinase signalling networks quantifies receptor-specific potencies of PI3K- and Ras-dependent ERK activation. Biochemical Journal. 2012;441(1):77-85. doi: 10.1042/bj20110833.

8. Cirit M, Wang CC, Haugh JM. Systematic quantification of negative feedback mechanisms in the extracellular signal-regulated kinase (ERK) signaling network. The Journal of biological chemistry. 2010;285(47):36736-44. Epub 2010/09/18. doi: 10.1074/jbc.M110.148759. PubMed PMID: 20847054; PubMed Central PMCID: PMCPmc2978602.

9. Derkinderen P, Valjent E, Toutant M, Corvol JC, Enslen H, Ledent C, et al. Regulation of extracellular signal-regulated kinase by cannabinoids in hippocampus. The Journal of neuroscience : the official journal of the Society for Neuroscience. 2003;23(6):2371-82. Epub 2003/03/27. PubMed PMID: 12657697.

10. Finch AR, Caunt CJ, Perrett RM, Tsaneva-Atanasova K, McArdle CA. Dual specificity phosphatases 10 and 16 are positive regulators of EGF-stimulated ERK activity: Indirect regulation of ERK signals by JNK/p38 selective MAPK phosphatases. Cellular Signalling. 2012;24(5):1002-11. doi: http://dx.doi.org/10.1016/j.cellsig.2011.12.021.

11. Hatakeyama M, Kimura S, Naka T, Kawasaki T, Yumoto N, Ichikawa M, et al. A computational model on the modulation of mitogen-activated protein kinase (MAPK) and Akt pathways in heregulin-induced ErbB signalling. The Biochemical journal. 2003;373(Pt 2):451-63. Epub 2003/04/15. doi: 10.1042/bj20021824. PubMed PMID: 12691603; PubMed Central PMCID: PMCPmc1223496.

12. Heitzler D, Durand G, Gallay N, Rizk A, Ahn S, Kim J, et al. Competing G protein‐coupled receptor kinases balance G protein and β‐arrestin signaling. Molecular Systems Biology. 2012;8(1). doi: 10.1038/msb.2012.22.

13. Hornberg JJ, Binder B, Bruggeman FJ, Schoeberl B, Heinrich R, Westerhoff HV. Control of MAPK signalling: from complexity to what really matters. Oncogene. 2005;24(36):5533-42. doi: http://www.nature.com/onc/journal/v24/n36/suppinfo/1208817s1.html.

14. Kamioka Y, Yasuda S, Fujita Y, Aoki K, Matsuda M. Multiple decisive phosphorylation sites for the negative feedback regulation of SOS1 via ERK. The Journal of biological chemistry. 2010;285(43):33540-8. Epub 2010/08/21. doi: 10.1074/jbc.M110.135517. PubMed PMID: 20724475; PubMed Central PMCID: PMCPmc2963383.

15. Kholodenko BN. Negative feedback and ultrasensitivity can bring about oscillations in the mitogen-activated protein kinase cascades. European journal of biochemistry / FEBS. 2000;267(6):1583-8. Epub 2000/03/11. PubMed PMID: 10712587.

16. Kiyatkin A, Aksamitiene E, Markevich NI, Borisov NM, Hoek JB, Kholodenko BN. Scaffolding Protein Grb2-associated Binder 1 Sustains Epidermal Growth Factor-induced Mitogenic and Survival Signaling by Multiple Positive Feedback Loops. Journal of Biological Chemistry. 2006;281(29):19925-38. doi: 10.1074/jbc.M600482200.

17. Kuhn C, Prasad KV, Klipp E, Gennemark P. Formal representation of the high osmolarity glycerol pathway in yeast. Genome informatics International Conference on Genome Informatics. 2010;22:69-83. Epub 2010/03/20. PubMed PMID: 20238420.

18. Li H, Ung CY, Ma XH, Li BW, Low BC, Cao ZW, et al. Simulation of crosstalk between small GTPase RhoA and EGFR-ERK signaling pathway via MEKK1. Bioinformatics. 2009;25(3):358-64. doi: 10.1093/bioinformatics/btn635.

19. Nakakuki T, Birtwistle MR, Saeki Y, Yumoto N, Ide K, Nagashima T, et al. Ligand-Specific c-Fos Expression Emerges from the Spatiotemporal Control of ErbB Network Dynamics. Cell. 2010;141(5):884-96. doi: http://dx.doi.org/10.1016/j.cell.2010.03.054.

20. Nakayama K, Satoh T, Igari A, Kageyama R, Nishida E. FGF induces oscillations of Hes1 expression and Ras/ERK activation. Current biology : CB. 2008;18(8):R332-4. Epub 2008/04/24. doi: 10.1016/j.cub.2008.03.013. PubMed PMID: 18430630.

21. Purvis J, Ilango V, Radhakrishnan R. Role of Network Branching in Eliciting Differential Short-Term Signaling Responses in the Hypersensitive Epidermal Growth Factor Receptor Mutants Implicated in Lung Cancer. Biotechnology Progress. 2008;24(3):540-53. doi: 10.1021/bp070405o.

22. Qi Z, Ming Y, Yan L. Spatial distribution and dose–response relationship for different operation modes in a reaction–diffusion model of the MAPK cascade. Physical Biology. 2011;8(5):055004.

23. Santos SDM, Verveer PJ, Bastiaens PIH. Growth factor-induced MAPK network topology shapes Erk response determining PC-12 cell fate. Nat Cell Biol. 2007;9(3):324-30. doi: http://www.nature.com/ncb/journal/v9/n3/suppinfo/ncb1543_S1.html.

24. Sarma U, Ghosh I. Different designs of kinase-phosphatase interactions and phosphatase sequestration shapes the robustness and signal flow in the MAPK cascade. BMC Systems Biology. 2012;6:82-. doi: 10.1186/1752-0509-6-82. PubMed PMID: PMC3508828.

25. Sarma U, Ghosh I. Oscillations in MAPK cascade triggered by two distinct designs of coupled positive and negative feedback loops. BMC research notes. 2012;5:287. Epub 2012/06/15. doi: 10.1186/1756-0500-5-287. PubMed PMID: 22694947; PubMed Central PMCID: PMCPmc3532088.

26. Sasagawa S, Ozaki Y-i, Fujita K, Kuroda S. Prediction and validation of the distinct dynamics of transient and sustained ERK activation. Nat Cell Biol. 2005;7(4):365-73. doi: http://www.nature.com/ncb/journal/v7/n4/suppinfo/ncb1233_S1.html.

27. Schoeberl B, Eichler-Jonsson C, Gilles ED, Muller G. Computational modeling of the dynamics of the MAP kinase cascade activated by surface and internalized EGF receptors. Nat Biotech. 2002;20(4):370-5. doi: http://www.nature.com/nbt/journal/v20/n4/suppinfo/nbt0402-370_S1.html.

28. Shankaran H, Ippolito DL, Chrisler WB, Resat H, Bollinger N, Opresko LK, et al. Rapid and sustained nuclear–cytoplasmic ERK oscillations induced by epidermal growth factor. Molecular Systems Biology. 2009;5:332-. doi: 10.1038/msb.2009.90. PubMed PMID: PMC2824491.

29. Sulpice E, Bryckaert M, Lacour J, Contreres J-O, Tobelem G. Platelet factor 4 inhibits FGF2-induced endothelial cell proliferation via the extracellular signal–regulated kinase pathway but not by the phosphatidylinositol 3–kinase pathway. Blood. 2002;100(9):3087-94. doi: 10.1182/blood.V100.9.3087.

30. Vasudevan HN, Mazot P, He F, Soriano P. Receptor tyrosine kinases modulate distinct transcriptional programs by differential usage of intracellular pathways. eLife. 2015;4:e07186. doi: 10.7554/eLife.07186. PubMed PMID: PMC4450512.

31. Vetterkind S, Saphirstein RJ, Morgan KG. Stimulus-specific activation and actin dependency of distinct, spatially separated ERK1/2 fractions in A7r5 smooth muscle cells. PloS one. 2012;7(2):e30409. Epub 2012/03/01. doi: 10.1371/journal.pone.0030409. PubMed PMID: 22363435; PubMed Central PMCID: PMCPmc3283592.

32. Wang CC, Cirit M, Haugh JM. PI3K-dependent cross-talk interactions converge with Ras as quantifiable inputs integrated by Erk. Mol Syst Biol. 2009;5:246. Epub 2009/02/20. doi: 10.1038/msb.2009.4. PubMed PMID: 19225459; PubMed Central PMCID: PMCPmc2657535.

33. Wei P, Wong WW, Park JS, Corcoran EE, Peisajovich SG, Onuffer JJ, et al. Bacterial virulence proteins as tools to rewire kinase pathways in yeast and immune cells. Nature. 2012;488(7411):384-8. doi: http://www.nature.com/nature/journal/v488/n7411/abs/nature11259.html#supplementary-information.

34. Yu RC, Pesce CG, Colman-Lerner A, Lok L, Pincus D, Serra E, et al. Negative feedback that improves information transmission in yeast signalling. Nature. 2008;456(7223):755-61. Epub 2008/12/17. doi: 10.1038/nature07513. PubMed PMID: 19079053; PubMed Central PMCID: PMCPmc2716709.
